# From LLM narratives to parameterized cooperation policies in multi-agent systems

**DOI:** 10.3389/frai.2026.1820827

**Published:** 2026-07-10

**Authors:** J. de Curtò, I. de Zarzà, Jordi Cabot, Pietro Manzoni, Carlos T. Calafate

**Affiliations:** 1 Department of Computer Applications in Science & Engineering, BARCELONA Supercomputing Center, Barcelona, Spain; 2 Escuela Técnica Superior de Ingeniería (ICAI), Universidad Pontificia Comillas, Madrid, Spain; 3 Estudis d‘Informàtica, Multimèdia i Telecomunicació, Universitat Oberta de Catalunya, Barcelona, Spain; 4 Human Centered AI, Data & Software, LUXEMBOURG Institute of Science and Technology, Esch-sur-Alzette, Luxembourg; 5 Interdisciplinary Centre for Security, Reliability and Trust (SNT), Université du Luxembourg, Esch-sur-Alzette, Luxembourg; 6 Departamento de Informática de Sistemas y Computadores, Universitat Politècnica de València, València, Spain

**Keywords:** cooperative equilibria, evolutionary game theory, large language models, multi-agent systems, network influence diffusion, non-stationary adaptation, parameterized policy compilation, policy interpretability

## Abstract

**Introduction:**

Large language models (LLMs) can generate persuasive narratives that shift agent behavior in multi-agent systems, but deploying raw, unstructured text as an influence mechanism offers no formal guarantees on effectiveness, interpretability, or controllability.

**Methods:**

We introduce the LLM Influence Compiler (LIC), a solver–critic pipeline that compiles natural-language cooperation directives into structured, parameterized influence policies defined over a five-field schema: network targeting, narrative theme, intensity, deployment timing, and compiler confidence. Each compiled policy is diffused through a network of numerical agents via an exposure model incorporating fatigue decay, susceptibility heterogeneity, and backlash. Evaluation spans nine controlled experiment blocks comprising more than 200 simulation runs across four topologies (Barabási–Albert, small-world, Erdős–Rényi, modular SBM), four network sizes (*n* ∈ {80, 160, 320, 640}), five LLM backbones, and two non-stationary perturbations.

**Results:**

Compiled policies raise the mean cooperation rate to 0.826 ± 0.010 (95% bootstrap CI [0.819, 0.834]), an 8.6% relative improvement over the unstructured baseline (0.760 ± 0.005, Mann–Whitney *p*_Bonf_ = 0.040, Cohen's *d* = 7.90). A controlled decomposition attributes the gain to network-aware targeting (*ca*. +4.5 pp), dose calibration (+1.9 pp), and an intervention floor (+1.5 pp); the residual contribution of full LLM compilation over a hand-coded rule with the same parameters is statistically indistinguishable from zero under stationary conditions. Under structural non-stationarity (mid-simulation graph rewire), the LLM-mediated re-deployment architecture significantly outperforms a frozen rule (paired t-test *p* < 0.001, paired Cohen's *d* = 2.27, 8/8 seeds). Adversarial stress testing confirms that the critic correctly flags 20/20 risky policies as high risk and rejects them, while passing a moderate-baseline policy at medium risk in all 5/5 trials.

**Discussion:**

The compiler abstraction converts an opaque generative process into a decomposable, auditable policy object whose components can be independently attributed, compared, and governed, laying the groundwork for constitutional oversight of LLM-mediated influence in artificial societies.

## 1 Introduction and related work

The capacity of large language models (LLMs) to generate contextually rich, persuasive natural-language text has opened a new design space for steering multi-agent systems toward desired collective outcomes (Park et al., 2023; Guo et al., 2024; Xi et al., 2023). Rather than hand-coding incentive structures or reward signals, a system designer can now instruct an LLM to produce a narrative, a motivational appeal, a framing of collective benefit, a reputational cue, and broadcast it to a population of agents whose cooperation probabilities update in response (de Curtò and de Zarzà, 2025; Piatti et al., 2024). Recent work has demonstrated that such LLM-mediated influence can meaningfully shift cooperation rates in iterated social dilemmas played on networks (Akata et al., 2023; Fan et al., 2024; Fontana et al., 2024), and that grounding LLM advice within evolutionary cooperation theory yields stable equilibria unattainable by either mechanism alone (de Zarzà et al., 2023).

Nevertheless, the very expressiveness that makes LLMs effective influence generators, also makes them opaque ones. When a model produces a paragraph urging agents to cooperate, several critical questions remain unanswered: Which agents should receive the message? Should the framing appeal to moral duty, economic self-interest, or group identity? How intense should the persuasive pressure be, and for how long? What happens when agents are overexposed? Do they comply, or do they recoil? In the absence of structure, the influence mechanism is a black box: effective on average, perhaps, but uninterpretable in its components, uncontrollable in its dosage, and unauditable in its downstream effects.

A clarifying note on terminology is warranted before proceeding. In what follows, “agents” refers to numerical entities whose cooperation decisions are governed by the logistic function of Equation 7; the LLM operates exclusively as a centralized policy compiler, not as the agent population itself. This isolates the contribution of structured policy compilation from the orthogonal question of how LLM-powered agents would reason about narrative content, an extension we discuss in Section 5. Throughout the paper, the theme multipliers μ(θ) should therefore be understood as exogenous dose factors stipulated to model the empirical observation that hybrid framings tend to outperform single-register appeals (Perc et al., 2017), rather than as predictions about how an LLM-powered agent would internalize narrative content.

This paper introduces the LLM Influence Compiler (LIC), a formal framework that addresses these limitations by *compiling* natural-language influence directives into structured, parameterized policy objects. The core insight is that the space of possible influence interventions, though expressed in the infinite-dimensional medium of natural language, can be projected onto a finite-dimensional policy schema whose fields correspond to actionable, independently tunable dimensions of intervention design. We define an Influence Policy Language (IPL) consisting of five fields:

- Theme (moral | economic | identity | hybrid): the narrative framing of the cooperative appeal;
- Intensity ([0, 1]): the strength of the persuasive signal;
- Target (hubs | bridges | periphery | random): which network positions receive direct exposure;
- Timing (periodic | burst): the deployment cadence;
- Confidence ([0, 1]): the compiler's self-assessed reliability of the policy.

A *solver–critic* pipeline compiles a population state snapshot into a concrete IPL instance: the solver proposes a policy optimizing for cooperation gain, and the critic evaluates it for polarization risk, backlash potential, and exposure equity, adjusting the intensity when necessary. The resulting policy is then executed through a network diffusion model that incorporates three mechanisms absent from naive broadcast approaches: *fatigue decay*, which prevents indefinite exposure accumulation; *susceptibility heterogeneity*, which reflects the empirical observation that agents differ in their responsiveness to persuasion; and *backlash mechanics*, which impose a cooperation penalty when exposure exceeds a threshold, modeling the well-documented phenomenon of reactance to perceived manipulation (Perc et al., 2017; Nowak, 2006).

The compiler abstraction yields three concrete benefits over unstructured LLM influence.

First, effectiveness. By allowing the LLM to reason about targeting strategy and narrative framing as explicit degrees of freedom rather than entangling them in free-form prose, compiled policies achieve a 6.6 ± 1.0 percentage-point improvement (mean ± s.d. across five seeds, 95% bootstrap CI on the gap [4.7, 8.5], Mann–Whitney *p*_Bonf_ = 0.040, Cohen's *d* = 7.90) over an unstructured narrative baseline. This advantage is robust across four network topologies (Barabási–Albert, small-world, Erdős–Rényi, modular SBM), persists undiminished as the population scales from 80 to 640 agents, and is reproduced with comparable magnitude across four large LLM backbones (Llama-3.3-70B, Qwen3-235B, Hermes-4-70B, DeepSeek-V3.2; mean gap +0.064). Llama-3.1-8B underperforms with a gap of +0.011, indicating a model-capability threshold for compiler-style structured reasoning. The advantage decomposes into a 2.2× dose factor (the compiler selects intensity 0.55 and confidence 0.80 vs. the unstructured defaults of 0.40 and 0.50) and a structural targeting factor: the compiler's hub-targeted seeding produces 4.5× higher average network exposure than random targeting, because high-degree nodes efficiently redistribute their dose to neighbors through gradient-based diffusion. Counterintuitively, hub targeting converges to *lower* final exposure inequality (Gini 0.15) than random targeting (Gini 0.23), because the steep exposure gradients around hubs drive rapid outward diffusion that self-corrects the initial concentration.

Second, interpretability through decomposition. Because each policy is a structured record with named fields, we can attribute cooperation outcomes to individual policy components. A controlled ablation holding all fields constant except targeting reveals that structurally informed strategies, targeting network hubs (high degree) or bridges (high betweenness centrality), yield cooperation rates of 0.828, vs. 0.779 for periphery targeting and 0.783 for random selection. We further show that hubs and bridges produce *numerically identical* outcomes at every timestep in the Barabási–Albert topology, implying that the cheaper degree-based selection is a perfect substitute for betweenness computation. At the agent level, the mechanism is quantified by the exposure redistribution ratio: under hub targeting, targeted nodes accumulate only 1.5× the exposure of non-targeted nodes (efficient diffusion), whereas under periphery targeting the ratio is 3.8× (trapped exposure). A parallel ablation over narrative themes shows that framing choice contributes at most 0.7 percentage points of variation, a 7.4× asymmetry favoring targeting. Notably, the compiler partially compensates for weaker theme multipliers by autonomously increasing intensity, compressing the effective dose range and demonstrating an emergent form of closed-loop control. The finding that *where* matters far more than *how* would be invisible without the decomposability afforded by structured policies.

Third, controllability and governability. A parameterized policy can be inspected, constrained, and filtered before deployment. One can impose constitutional rules, for example, forbid misleading claims, cap intensity at 0.8, require exposure equity below a Gini threshold, and verify compliance mechanically. This is impossible when the influence mechanism is an unconstrained paragraph of LLM-generated text. The compiler thus lays the architectural foundation for governance frameworks that ensure influence is not only effective but is also ethical.

### 1.1 Relationship to prior work

Our work sits at the intersection of three research streams.

#### 1.1.1 LLM-based multi-agent systems

The generative agents paradigm (Park et al., 2023) demonstrated that LLM-driven agents can exhibit emergent social behavior, and subsequent work has scaled this to multi-agent debate (Du Y. et al., 2024; Liang et al., 2024), role-playing societies (Li et al., 2024), and cooperative dilemmas (Piatti et al., 2024). These systems uniformly treat LLM output as an unstructured signal, the agent receives text and updates its behavior accordingly. We formalize the intermediate representation between the LLM and the agent population, introducing a compilation step that converts free-form text into a structured policy.

A growing body of work uses LLMs to simulate artificial societies in which agents themselves are LLM-powered (Park et al., 2023; Gao et al., 2023; Kaiya et al., 2023; Mou et al., 2026). These systems demonstrate emergent norms, opinion dynamics, and coordination behavior, but agent-level LLM reasoning remains the unit of analysis and the LLM mediates both perception and action. Our framework occupies a complementary position: the LLM is centralized as a policy compiler, while the agent population is numerical. This separation isolates the contribution of structured policy compilation from confounds introduced by LLM-driven agent heterogeneity (instruction-following variance, prompt sensitivity, reasoning failures), and admits closed-form analysis of diffusion and cooperation dynamics that would be opaque if every agent were a black-box LLM.

#### 1.1.2 Evolutionary cooperation on networks

The theoretical foundations of cooperation in structured populations are well-established. (Nowak 2006) five rules identify the mechanisms: kin selection, direct and indirect reciprocity, spatial selection, group selection, that sustain cooperation against defection. (Santos and Pacheco 2005) showed that scale-free network topology amplifies cooperation by concentrating interactions around hubs, and (Ohtsuki et al. 2006) derived the *b*/*c* > *k* rule for cooperation on regular graphs. (Szabó and Fáth 2007) provide a comprehensive treatment of evolutionary games on graphs, including the Fermi update rule we employ for social learning. (Perc et al. 2017) offer a statistical-physics perspective on human cooperation that motivates our treatment of polarization as variance in cooperation probability. Our contribution is to embed LLM-compiled influence within this framework, treating the compiler output as an exogenous intervention whose network-mediated diffusion follows established evolutionary dynamics.

Recent work couples opinion dynamics with cooperation games on networks, showing that opinion–game feedback can sustain or destabilize equilibria (Zino and Cao, 2021; Anagnostopoulos et al., 2022). Reinforcement-learning-driven adaptive networks, where agents simultaneously update strategies and rewire ties, have also been explored (Mastrandrea et al., 2024; Du C. et al., 2024). The LIC framework can be read as an external coordination signal complementary to such mechanisms: rather than agents learning who to interact with, an external compiler decides where to apply directed exposure. Our R8b experiment (Section 4.14) demonstrates that this external loop provides measurable adaptive value precisely when the network's structural assumptions shift, the regime in which adaptive-network mechanisms are most relevant.

#### 1.1.3 Persuasion resistance and information overload

The backlash term in our diffusion model is grounded in the empirical literature on psychological reactance (Brehm and Brehm, 2013; Knowles and Linn, 2004): when audiences perceive manipulation or exceed an exposure threshold, compliance reverses and attitudes can move *against* the persuasive intent. Our backlash threshold *e*_β_ and penalty rate ρ operationalize this at the population level. The fatigue decay term, modeled as multiplicative exposure decay between deployment events, draws on the information-overload literature (Eppler and Mengis, 2004; Schmitt et al., 2018), which documents that audiences habituate to sustained messaging campaigns and that effective influence saturates at a sub-maximal dose. These two mechanisms together prevent the trivial outcome in which arbitrarily strong influence drives cooperation arbitrarily high; the diffusion model thus admits a non-trivial Pareto frontier between cooperation gain, polarization, and equity (Section 4.5).

#### 1.1.4 Influence, persuasion, and diffusion

The study of how information and behavior spread through networks has a long history in sociology, epidemiology, and computational social science (Newman, 2003; Watts and Strogatz, 1998). The innovation here is not diffusion itself but the *source*: an LLM that can be prompted to generate influence with specific properties, and a compilation layer that ensures those properties are explicit, bounded, and auditable. The backlash and fatigue mechanisms in our diffusion model are inspired by the empirical literature on persuasion resistance and information overload, adapted to the multi-agent simulation setting.

### 1.2 Contributions

The specific contributions of this paper are:

1. The Influence Policy Language (IPL), a five-field parameterized schema that formalizes the design space of LLM-generated influence interventions in multi-agent societies.
2. The LLM Influence Compiler, a solver–critic pipeline that compiles population state snapshots into IPL-conformant policies. We demonstrate that the critic is well-calibrated in production (100% medium verdict on solver-generated policies across 360 calls, with zero parsing failures) and responsive under adversarial stress (20/20 hand-crafted risky policies flagged high and rejected, mean intensity attenuation −0.77).
3. An enhanced diffusion model incorporating fatigue decay, susceptibility heterogeneity, and backlash mechanics, with self-correcting equity dynamics under hub-targeted seeding.
4. A comprehensive multi-seed experimental evaluation with bootstrap confidence intervals, Mann–Whitney significance testing under Bonferroni correction (*m* = 5), Cohen's *d* effect sizes, multi-topology validation (BA, WS, ER, modular SBM), multi-scale validation (*n* ∈ {80, 160, 320, 640}), and multi-model robustness (five LLM backbones).
5. A controlled policy decomposition attributing the compiled-vs.-unstructured gap to its components: intervention floor (+1.5 pp), dose calibration (+1.9 pp), network-aware targeting (+4.5 pp), and a residual “+ state information” component (+0.2 pp) that is statistically indistinguishable from zero under stationary conditions, motivating the non-stationary adaptation analysis.
6. A non-stationary adaptation analysis that resolves the apparent stationary equivalence: under structural perturbation (mid-simulation graph rewire, *n* = 8 seeds), the LLM-mediated re-deployment architecture significantly outperforms a frozen rule with identical static parameters (paired Wilcoxon *p* = 0.004, paired *t* *p* < 0.001, *d*_paired_ = 2.27). The compiler's adaptive value lies in re-resolving abstract policy language (hubs) against the *current* network state at each deploy, not in real-time policy parameter optimization.
7. A 7.4× targeting-to-framing attribution ratio, quantifying under controlled conditions a relationship long understood in network science (Kempe et al., 2003; Watts and Dodds, 2007) and demonstrating that the compiler autonomously selects the dominant component (hub targeting) under a free policy space.

The remainder of this paper is organized as follows. Section 2 presents the influence policy language, the compiler architecture, and the enhanced diffusion model. Section 3 describes the experimental setup and the full suite of ablation studies. Section 4 reports quantitative results across all conditions. Section 5 interprets the findings and discusses limitations. Finally, Section 6 concludes the paper, and refers to future work.

## 2 Methods

This section presents the four components of the LIC framework: the Influence Policy Language that defines the structured output space (Section 2.1), the solver–critic compiler that maps population state to policy instances (Section 2.2), the network model and targeting strategies that determine where influence is applied (Section 2.3), and the diffusion–cooperation model that governs how exposure propagates and translates into behavioral change (Section 2.4).

**Figure 1:**
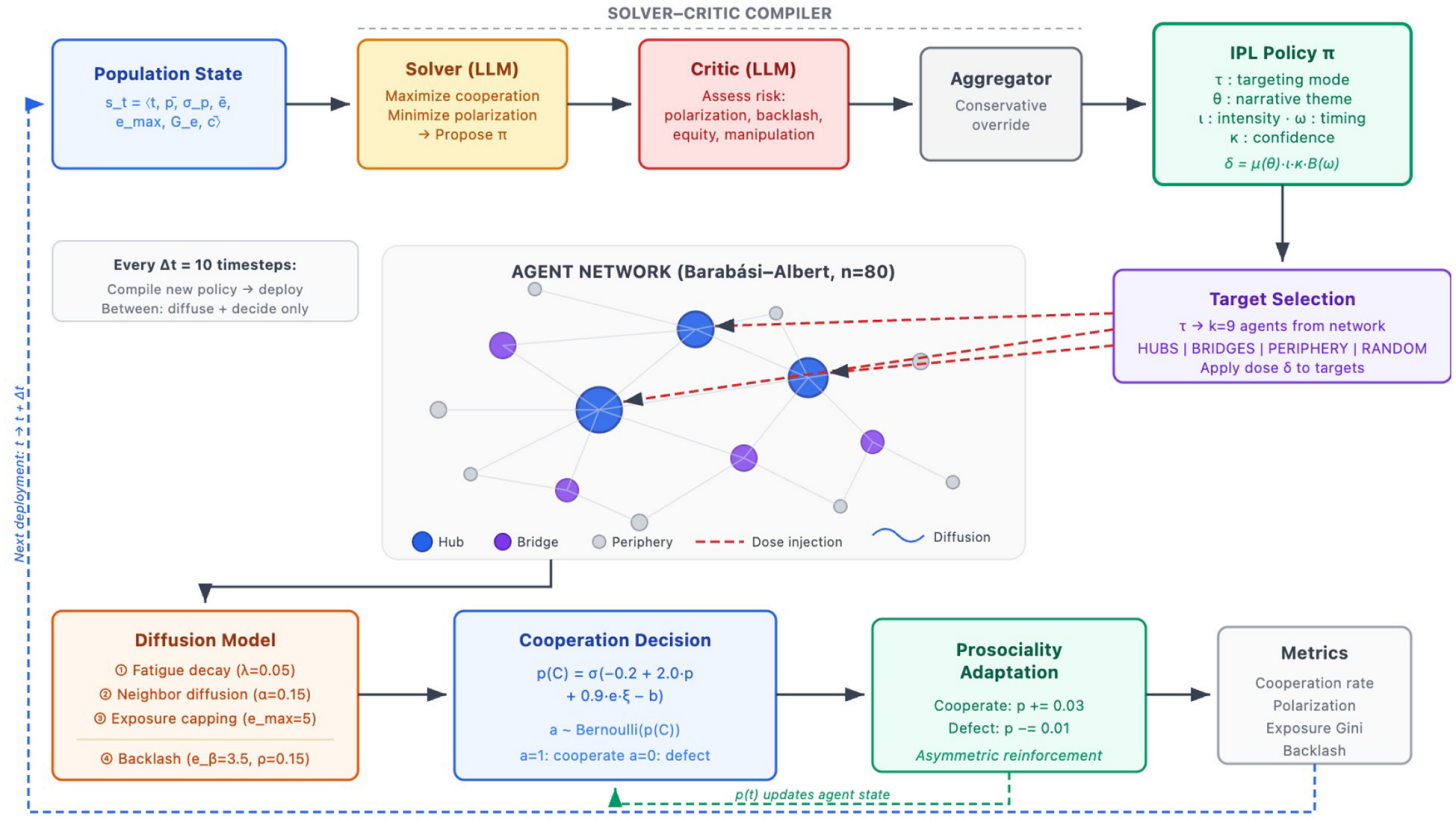
Architectural overview of the LIC framework. The solver–critic compiler (**top**) produces a structured IPL policy from a population state snapshot. The policy is deployed onto the agent network (**center**) via targeted dose injection, and exposure propagates through gradient-driven diffusion. The bottom row shows the per-timestep agent dynamics. The blue dashed path closes the feedback loop every Δ*t* = 10 timesteps.

Figure 1 provides an architectural overview of the complete pipeline. The top row shows the solver–critic compiler: the solver ingests a population state snapshot *s*_*t*_ and proposes a structured IPL policy π, which the critic evaluates for risk before the aggregator emits the final policy. The targeting module (right) maps the policy's targeting mode τ to *k* = 9 agents in the network and applies the effective dose δ. Between deployments, exposure propagates through the Barabási–Albert network via gradient-driven diffusion (center). At every timestep, agents make stochastic cooperation decisions through the logistic function of Equation 7 and update their prosociality via asymmetric reinforcement (bottom row). The resulting population metrics feed the next state snapshot, closing the compilation loop every Δ*t* = 10 timesteps.

### 2.1 Influence policy language

The central design decision of the LIC framework is to replace unconstrained natural-language influence with a finite-dimensional, typed policy object. We define the Influence Policy Language (IPL) as a structured record, shown in Equation 1:


$$\begin{array}{l} \pi =\langle \tau ,\theta ,\iota ,\omega ,\kappa \rangle \end{array}$$ (1)

where each field takes values from a restricted domain:

- τ ∈ {hubs, bridges, periphery, random}: the *targeting mode*, specifying which network positions receive direct exposure (Section 2.3);
- θ ∈ {moral, economic, identity, hybrid}: the *narrative theme*, determining the framing of the cooperative appeal;
- ι ∈ [0, 1]: the *intensity*, controlling the magnitude of the persuasive signal;
- ω ∈ {burst, periodic}: the *timing mode*, where burst applies the intervention for *B* consecutive sub-steps and periodic applies it once;
- κ ∈ [0, 1]: the *compiler confidence*, a self-assessed reliability score that modulates the effective dose.

Each compiled policy additionally carries a free-text *reasoning* field that records the LLM's justification for its choices, providing an interpretability trace without affecting the simulation dynamics.

#### 2.1.1 Theme effect multipliers

Narrative framing modulates the effective dose through a theme-dependent multiplier μ(θ). We set μ(moral) = 1.15, μ(economic) = 1.05, μ(identity) = 1.10, and μ(hybrid) = 1.20. These weights reflect the empirical observation that hybrid framings that combine multiple appeal registers tend to be more persuasive than single-register appeals, while identity-based and moral framings outperform purely economic arguments in public-goods contexts (Perc et al., 2017). The effective dose delivered to each targeted agent at deployment time *t*_*d*_ is:


$$\begin{array}{l} \delta =\mu \left(\theta \right)\cdot \iota \cdot \kappa \cdot B\left(\omega \right). \end{array}$$ (2)

The timing factor *B*(ω) specifies the temporal delivery profile of the dose. Under ω =periodic, the effective dose δ is delivered to each targeted agent in a single timestep at the deploy event *t*_*d*_. Under ω =burst, the same effective dose is delivered as *L* = 3 consecutive sub-doses across timesteps [*t*_*d*_, *t*_*d*_ + *L* − 1], modeling a sustained micro-campaign rather than an instantaneous concentrated injection. We adopt this *sequential* interpretation rather than a 3× multiplier applied at *t*_*d*_. While both interpretations produce statistically indistinguishable final cooperation rates (0.906 ± 0.005 vs. 0.906 ± 0.006 across five seeds, Mann–Whitney *p* > 0.5), the multiplier variant generates 3.27× higher cumulative backlash because it briefly drives per-node exposure above the saturation threshold *e*_β_, whereas the sequential variant spreads the dose under the threshold. We report the empirical comparison in Section 4.11.

#### 2.1.2 Normalization and validation

Because the compiler's LLM backbone may produce out-of-range or malformed values, every policy undergoes a normalization step before execution. Categorical fields are matched case-insensitively against their valid sets, with defaults applied on failure (random for targeting, economic for theme, periodic for timing). Continuous fields are clipped to [0, 1]. This normalization layer ensures that the simulation is never disrupted by LLM generation errors, while the raw solver output is preserved in metadata for *post-hoc* analysis.

### 2.2 Solver–Critic influence compiler

The compiler transforms a population state snapshot into an IPL instance through a two-stage pipeline.

#### 2.2.1 Stage 1: Solver

The solver receives a JSON-formatted state snapshot *s*_*t*_ summarizing the current societal condition:


$$\begin{array}{l} s_{t}=\langle t,{\bar{p}}_{t},\sigma_{p,t},{ē}_{t},e_{t}^{\max},G_{e}\left(t\right),{\bar{c}}_{\text{recent}}\rangle \end{array}$$ (3)

where ${\bar{p}}_{t}$ and σ_*p,t*_ are the mean and standard deviation of agent prosociality, ē_*t*_ and $e_{t}^{\max}$ are the mean and maximum exposure levels, *G*_*e*_(*t*) is the Gini coefficient of the exposure distribution, and ${\bar{c}}_{\text{recent}}$ is the average cooperation rate over the preceding five timesteps. The solver is instructed via a system prompt to produce a JSON object conforming to the IPL schema, with an explicit mandate to maximize long-run cooperation while minimizing polarization and backlash risk. The raw LLM output is parsed and normalized into an InfluencePolicy instance.

#### 2.2.2 Stage 2: Critic

The critic receives both the solver's proposed policy and the state snapshot, and is instructed to evaluate the policy along four risk dimensions: polarization amplification, backlash potential, exposure inequity, and manipulation risk. Its output is a structured assessment containing an approval decision (boolean), a risk level (low, medium, or high), an adjusted intensity recommendation, and a textual explanation.

#### 2.2.3 Aggregation

The aggregator fuses the solver and critic signals according to the following rules:

- If the critic *rejects* the policy: intensity is halved and clipped to [0.1, 0.8], and confidence is reduced by 30%;
- If the critic assigns high risk: intensity is reduced by 30% and clipped to [0.1, 0.9];
- If the critic assigns medium risk: the critic's adjusted intensity is applied directly, clipped to [0.1, 1.0];
- If the critic assigns low risk: the solver's policy is used without modification.

This asymmetric aggregation implements a *conservative override* principle: under rejection or high risk the critic strictly attenuates the solver's proposal, and under medium risk it applies the critic's adjusted intensity directly. In practice, the critic consistently recommends equal or lower intensity than the solver across all conditions in our experiments, so the pipeline effectively errs on the side of caution when risk signals are detected.

#### 2.2.4 Fallback

If either stage produces unparseable output, the system falls back to a safe default policy: π_default_ = 〈random, economic, 0.5, periodic, 0.65〉. This ensures graceful degradation under LLM failure while preserving simulation continuity.

Algorithm 1 summarizes the complete compilation cycle.

Algorithm 1 **LIC Compilation Cycle**


Require:  Population state snapshot *s*_*t*_ (Equation 3)
Ensure:  Deployed influence policy π
1:  π_*s*_ ← solver(*s*_*t*_) ⊳ Propose IPL policy
2:  π_*s*_ ← normalize(π_*s*_) ⊳ Clip and validate fields
3:  (*d, r*, ι_adj_)←critic(π_*s*_, *s*_*t*_) ⊳ Risk assessment
4:  if *d* =reject **then**
5:   ι←clip(ι_*s*_/2, 0.1, 0.8); κ←0.7κ_*s*_
6:  else **if** *r* =high **then**
7:   ι←clip(0.7ι_*s*_, 0.1, 0.9)
8:  else **if** *r* =medium **then**
9:   ι←clip(ι_adj_, 0.1, 1.0)
10:  else⊳ low risk
11:   ι←ι_*s*_
12:  end **if**
13:  π←π_*s*_ with updated ι, κ
14:  δ←μ(θ)·ι·κ·*B*(ω) ⊳ Effective dose (Equation 2)
15:  Deploy(π, δ) to *k* agents selected by τ

### 2.3 Network model and targeting strategies

#### 2.3.1 Graph generation

Agents are embedded in a scale-free network generated by the Barabási–Albert preferential attachment model (Barabási and Albert, 1999) with *n* = 80 nodes and attachment parameter *m* = 3, yielding an average degree of approximately 6. This topology was chosen because scale-free networks are known to promote cooperation through hub-mediated amplification (Santos and Pacheco, 2005), creating a structured testbed in which targeting strategies have heterogeneous effects. Connectivity is enforced post-generation: any isolated component is bridged to the giant component by adding a single edge between a random node in each component.

#### 2.3.2 Targeting strategies

The targeting mode τ determines which fraction *f* = 0.12 of agents (i.e., *k* = ⌊0.12 × 80⌋ = 9 agents) receive direct exposure at each deployment. We implement four strategies:

- hubs: the *k* agents with highest degree, leveraging their connectivity to maximize one-hop diffusion reach;
- bridges: the *k* agents with highest betweenness centrality (Newman, 2003), targeting structural bottlenecks that mediate information flow between communities;
- periphery: the *k* agents with lowest degree, representing an alternative hypothesis that underconnected agents are underserved and thus most susceptible to influence;
- random: *k* agents selected uniformly at random, serving as a network-agnostic baseline.

The distinction between hubs and bridges is particularly important in scale-free networks. While hubs have many connections, bridges may have moderate degree but occupy topologically critical positions. Our experiments test whether this structural distinction translates into differential influence effectiveness.

### 2.4 Diffusion and cooperation model

The diffusion model governs how exposure propagates through the network after deployment, and how accumulated exposure converts into cooperation decisions. We introduce three mechanisms that extend naive broadcast diffusion: fatigue decay, gradient-driven neighbor diffusion, and exposure capping.

It is important to note that the agents in our model are not LLM-based: they are numerical entities whose cooperation decisions are governed by the logistic function of Equation 7. The only LLM in the system is the compiler itself, which operates as a centralized policy optimizer with access to aggregate population statistics. Agents do not receive or interpret natural-language messages; the compiled policy manifests as a numerical dose applied to the exposure state of targeted agents. This design isolates the contribution of structured policy compilation from the separate question of how LLM-powered agents would process narrative content, an extension we discuss in Section 5.

#### 2.4.1 Exposure dynamics

Let *e*_*o*_(*t*) ∈ [0, *e*_max_] denote the exposure of agent *o* at time *t*, where *e*_max_ is a hard cap imposing diminishing returns. The exposure update comprises three stages applied at every timestep:

*(1) Fatigue decay*. Exposure decays multiplicatively at rate λ, preventing indefinite accumulation:


$$\begin{array}{l} {ẽ}_{o}\left(t\right)=\left(1-\lambda \right)e_{o}\left(t\right) \end{array}$$ (4)

*(2) Neighbor diffusion*. Exposure flows from high-exposure to low-exposure neighbors, proportional to the local gradient:


$$\begin{array}{l} e_{o}\left(t+1\right)={ẽ}_{o}\left(t\right)+\alpha \cdot \max\left(0,\frac{1}{\left|N_{o}\right|}\underset{z\in N_{o}}{\sum }e_{z}\left(t\right)-e_{o}\left(t\right)\right) \end{array}$$ (5)

where $N_{o}$ is the neighbor set of agent *o* in *G*, and α is the diffusion rate. The max(0, ·) operator ensures that diffusion is strictly uphill: agents receive exposure from more-exposed neighbors but do not lose exposure to less-exposed ones. This models the observation that influence propagates outward from seeded nodes but does not reverse.

*(3) Capping*. After diffusion, exposures are clipped: *e*_*o*_(*t* + 1) ← min(*e*_*o*_(*t* + 1), *e*_max_).

#### 2.4.2 Backlash mechanics

When an agent's exposure exceeds a backlash threshold *e*_β_, a cooperation penalty is incurred:


$$\begin{array}{l} b_{o}\left(t\right)=\rho \cdot \max\left(0,e_{o}\left(t\right)-e_{\beta }\right) \end{array}$$ (6)

where ρ is the backlash penalty rate. This implements a saturation effect: moderate exposure promotes cooperation, but overexposure triggers reactance that actively reduces cooperation probability.

#### 2.4.3 Cooperation decision

Each agent's cooperation probability is computed via a logistic function that integrates prosociality, exposure, susceptibility, and backlash:


$$\begin{array}{l} p{\left(\text{C}\right)}_{o}\left(t\right)=\sigma \left(\underset{\text{bias}}{\underset{︸}{\beta_{0}}}+\underset{\text{prosociality}}{\underset{︸}{\beta_{p}\cdot p_{o}\left(t\right)}}+\underset{\text{exposure}}{\underset{︸}{\beta_{e}\cdot e_{o}\left(t\right)\cdot \xi_{o}}}-\underset{\text{backlash}}{\underset{︸}{b_{o}\left(t\right)}}\right) \end{array}$$ (7)

where *p*_*o*_(*t*) ∈ (0, 1) is the agent's prosociality trait, ξ_*o*_ ∈ (0, 1) is a static susceptibility parameter drawn once at initialization, σ(*x*) = 1/(1 + *e*^−*x*^), and β_0_, β_*p*_, β_*e*_ are the bias, prosociality weight, and exposure weight respectively. The negative bias ensures that without exposure, agents with moderate prosociality cooperate at a rate consistent with empirical observations in public-goods games.

The agent then draws a Bernoulli action: *a*_*o*_(*t*)~Bernoulli(*p*(C)_*o*_(*t*)), where *a*_*o*_ = 1 denotes cooperation and *a*_*o*_ = 0 denotes defection.

#### 2.4.4 Modeling rationale

The terms in Equations 4–7 are grounded in established literatures rather than chosen arbitrarily. *Multiplicative fatigue decay* (Equation 4) models habituation in persuasion psychology and information-overload dynamics in digital media (Eppler and Mengis, 2004; Schmitt et al., 2018), where audiences habituate to sustained messaging at a rate roughly proportional to current attention level. *Gradient-based diffusion* (Equation 5) inherits its monotone-uphill structure from epidemiological compartmental models and opinion-dynamics literature (Newman, 2003); the max(0, ·) operator implements the empirical asymmetry that influence propagates outward from seeded nodes but does not reverse direction once received. *Asymmetric prosociality update* (γ^+^ > γ^−^, Equation 8) reflects the empirical observation that cooperative dispositions accumulate slowly through repeated cooperation but are not erased by isolated defections, an asymmetry consistent with loss-aversion and habit-formation findings in behavioral economics. The *backlash* term (Equation 6) operationalizes psychological reactance (Brehm and Brehm, 2013; Knowles and Linn, 2004) at the population level: exposure above *e*_β_ triggers a cooperation penalty proportional to the excess. We *clip* rather than rescale exposure (the cap step in Equation 5) because clipping preserves the linear interpretation of intensity within the unsaturated range; rescaling would compress the absolute dose interpretation and break the additive decomposition reported in Section 4.6.

#### 2.4.5 Prosociality adaptation

Prosociality is not static but adapts in response to the agent's own behavior, creating a feedback loop between action and disposition:


$$\begin{array}{l} p_{o}\left(t+1\right)=\text{clip}\left[p_{o}\left(t\right)+\gamma^{+}\left(a_{o}\left(t\right)-p_{o}\left(t\right)\right)-\gamma^{-}\left(1-a_{o}\left(t\right)\right),0.01,0.99\right] \end{array}$$ (8)

Cooperators experience a gentle drift toward higher prosociality (self-reinforcing cooperation), while defectors incur a prosociality penalty. The asymmetric rates (γ^+^ > γ^−^) ensure that sustained cooperation gradually shifts the population toward a more prosocial equilibrium, while occasional defection does not immediately erase accumulated prosociality.

#### 2.4.6 Polarization

We measure polarization as the variance of the cooperation probability distribution across agents:


$$\begin{array}{l} \text{Pol}\left(t\right)=\text{Var}\left[p{\left(\text{C}\right)}_{o}\left(t\right)\right]=\frac{1}{n}\overset{n}{\underset{o=1}{\sum }}{\left(p{\left(\text{C}\right)}_{o}\left(t\right)-\bar{p\left(\text{C}\right)}\left(t\right)\right)}^{2} \end{array}$$ (9)

This metric captures the degree to which the population has split into committed cooperators and committed defectors, with lower values indicating more consensus.

#### 2.4.7 Exposure equity

We track the Gini coefficient of the exposure vector ***e***(*t*) as a fairness measure. A Gini of 0 indicates perfectly uniform exposure; a Gini approaching 1 indicates that exposure is concentrated on a few agents. Targeting strategies that produce low Gini are preferable from an equity standpoint, as they ensure that influence is distributed broadly rather than concentrated on a privileged subpopulation.

### 2.5 Simulation protocol

The simulation runs for *T* = 100 discrete timesteps. At initialization, each agent *o* is assigned a prosociality $p_{o}\left(0\right)\sim N\left(0.45,0.12^{2}\right)$ and a susceptibility $\xi_{o}\sim N\left(0.55,0.15^{2}\right)$, both clipped to (0.01, 0.99). Exposure is initialized to zero for all agents.

Policy deployment occurs every Δ*t* = 10 timesteps. At each deployment event, the system constructs a state snapshot *s*_*t*_ (Equation 3), invokes the compiler to produce a policy π_*t*_, selects *k* = 9 target agents according to the targeting mode τ, and applies the effective dose δ (Equation 2) to the targets. Between deployments, the simulation proceeds with diffusion, backlash computation, cooperation decisions, and prosociality adaptation but no new influence injection.

The LLM backbone is Meta's Llama-3.3-70B-Instruct, accessed via the Nebius AI Studio API at temperature 0.2 with a maximum of 350 tokens per call. All LLM responses are SHA1-cached to ensure reproducibility across runs and reduce API cost. The simulation is fully seeded (random, NumPy) for deterministic agent initialization and targeting.

For convenience, Table 1 collects all symbols introduced in this section with their definitions and domains.

**Table 1:** Summary of notation.

Symbol	Meaning	Domain
Influence policy language		
π	Compiled policy instance	IPL record
τ	Targeting strategy	{hubs, bridges, periphery, random}
θ	Narrative theme	{moral, economic, identity, hybrid}
ι	Intensity	[0, 1]
ω	Timing mode	{burst, periodic}
κ	Compiler confidence	[0, 1]
μ(θ)	Theme effect multiplier	ℝ^+^
δ	Effective dose	ℝ^+^
Agent state		
*p*_*o*_(*t*)	Prosociality of agent *o*	(0.01, 0.99)
ξ_*o*_	Susceptibility of agent *o*	(0.01, 0.99)
*e*_*o*_(*t*)	Exposure of agent *o*	[0, *e*_max_]
*b*_*o*_(*t*)	Backlash penalty	ℝ_≥0_
*p*(C)_*o*_(*t*)	Cooperation probability	(0, 1)
*a*_*o*_(*t*)	Cooperation action	{0, 1}
Network and diffusion		
*G*	Agent network	BA graph
$N_{o}$	Neighbor set of agent *o*	⊆*V*(*G*)
α	Diffusion rate	ℝ^+^
λ	Fatigue decay rate	(0, 1)
*e*_max_	Exposure cap	ℝ^+^
*e*_β_	Backlash threshold	ℝ^+^
ρ	Backlash penalty rate	ℝ^+^
Cooperation model		
β_0_	Bias	ℝ
β_*p*_	Prosociality weight	ℝ^+^
β_*e*_	Exposure weight	ℝ^+^
γ^+^	Reinforcement rate	(0, 1)
γ^−^	Erosion rate	(0, 1)
Metrics		
Pol(*t*)	Polarization	ℝ_≥0_
*G*_*e*_(*t*)	Exposure Gini coefficient	[0, 1]
*s*_*t*_	Population state snapshot	JSON record

## 3 Experimental setup

We evaluate the LIC framework through six experiments organized around three questions: (1) Does compilation improve over unstructured influence? (2) Which policy components contribute most to cooperation gains? and (3) What are the trade-offs between targeting strategies? All experiments share a common infrastructure described below, with controlled variations isolating specific factors.

### 3.1 Common configuration

All experiments use the parameter settings summarized in Table 2. The population consists of *n* = 80 agents on a Barabási–Albert scale-free network with attachment parameter *m* = 3. Simulations run for *T* = 100 timesteps with policy deployment every Δ*t* = 10 steps, yielding 10 deployment events per run. At each deployment, *k* = ⌊0.12 × 80⌋ = 9 agents are selected as direct targets.

**Table 2:** Default simulation parameters.

Parameter	Symbol	Value
Population		
Number of agents	*n*	80
Network topology	—	Scale-free (BA)
Attachment parameter	*m*	3
Agent initialization		
Base prosociality	${\bar{p}}_{0}$	$N\left(0.45,0.12^{2}\right)$
Susceptibility	$\bar{\xi }$	$N\left(0.55,0.15^{2}\right)$
Simulation		
Timesteps	*T*	100
Deployment interval	Δ*t*	10
Target fraction	*f*	0.12
Burst length	*B*	3
Random seed	—	0
Diffusion model		
Diffusion rate	α	0.15
Fatigue decay rate	λ	0.05
Exposure cap	*e*_max_	5.0
Backlash threshold	*e*_β_	3.5
Backlash penalty rate	ρ	0.15
LLM backbone		
Model	—	Llama-3.3-70B-Instruct
Temperature	—	0.2
Max tokens	—	350
Cooperation model		
Bias	β_0_	−0.2
Prosociality weight	β_*p*_	2.0
Exposure weight	β_*e*_	0.9
Reinforcement rate	γ^+^	0.03
Erosion rate	γ^−^	0.01

Agent prosociality and susceptibility are initialized from truncated Gaussian distributions clipped to (0.01, 0.99). The random seed is fixed at 0 across all experiments to ensure identical agent initializations and network structures, so that observed differences are attributable solely to the experimental manipulation.

### 3.2 Experiment 1: compiled vs. unstructured influence

The primary experiment compares the full LIC pipeline (solver–critic compilation) against an *unstructured narrative baseline*. Both conditions use the same LLM backbone, agent population, and network.

In the compiled condition, the solver–critic pipeline generates a structured IPL policy at each deployment event, with all fields (targeting, theme, intensity, timing, confidence) determined by the LLM's reasoning over the population state snapshot. The critic evaluates risk and may attenuate intensity.

In the unstructured condition, the LLM generates a free-form motivational narrative (2–3 sentences of natural language) at each deployment event. Because this narrative lacks structured fields, it is mapped to a fixed low-precision policy with conservative defaults: random targeting, hybrid theme, intensity ι = 0.4, periodic timing, and confidence κ = 0.5. This design choice reflects the core thesis: without explicit compilation, the system has no mechanism to select targets intelligently, calibrate intensity, or choose a deployment cadence, it can only apply a uniform, moderate signal to randomly selected agents.

The specific default values 〈random, hybrid, ι = 0.4,periodic, κ = 0.5〉 are not tuned to flatter the compiled condition, and the paper's claims do not rest on the size of the compiled-vs.-unstructured gap. Three results establish this. First, the default is the operationalization of “absence of structured reasoning”: random targeting encodes no targeting intelligence, and mid-range intensity and confidence encode no calibration. The R1 decomposition (Section 4.6, Table 3) confirms this is not an arbitrarily weak point but a principled one: the unstructured condition (0.764) coincides *exactly* with static_random_low (0.764), the diffusion floor plus a minimal undirected intervention, rather than landing below it. Second, the gap is not an artifact of the chosen dose. The static_random_full condition assigns the unstructured policy the compiler's *own* dose (ι = 0.55, κ = 0.80) at random targeting and still reaches only 0.783, 4.5 pp below static_hub_full (0.828); no choice of the default's intensity or confidence closes the gap, because the gap is governed by targeting, not dose. A dedicated 5 × 5 sensitivity sweep of the default's intensity and confidence at random targeting (Section 4.7, Table 4) confirms this directly: across 25 parameterizations the unstructured baseline never exceeds 0.797, remaining at least 3.1 pp below static_hub_full even at doses larger than the compiler's own. Third, and decisively, the paper does not claim superiority over a strong hand-coded baseline: under multi-seed analysis (Section 4.8) compiled is statistically *indistinguishable* from static_hub_full, the strongest non-LLM rule (*d* = 0.18, *p*_Bonf_ = 1.00). The unstructured baseline therefore serves only to quantify the cost of *forgoing structured compilation entirely*; the framework's substantive contribution is the architectural adaptation result of Section 4.14, established against static_hub_full, not against the unstructured default. The decomposition design specifically exists so that no specific benchmark choice can drive the conclusions.

**Table 3:** R1 decomposition: attributing the compiled-vs.-unstructured gap to policy components.

Condition	Targeting	Dose (ι/κ)	State	${\bar{c}}_{\text{final}}$
no_intervention	—	—	—	0.749
static_random_low	Random	0.40 / 0.50	—	0.764
static_random_full	Random	0.55 / 0.80	—	0.783
static_hub_low	Hubs	0.40 / 0.50	—	0.783
static_hub_full	Hubs	0.55 / 0.80	—	0.828
blind_llm	LLM	LLM	No	0.786
compiled	LLM	LLM	Yes	**0.830**
unstructured	random	0.40 / 0.50	—	0.764

All conditions share identical agent initialization (*n* = 80, BA, seed = 0). Final cooperation ${\bar{c}}_{\text{final}}$ averaged over *t* ∈ [86, 100]. Bold value indicates the highest final cooperation rate ${\bar{c}}_{\text{final}}$ across all conditions.

**Table 4:** R1-1 unstructured-default sensitivity: final cooperation ${\bar{c}}_{\text{final}}$ over a 5 × 5 grid of the default's intensity ι and confidence κ, at random targeting (hybrid/periodic, *n* = 80, BA, seed = 0).

Intensity ι	Confidence κ				
	0.40	0.50	0.60	0.70	0.80
0.30	0.759	0.761	0.762	0.764	0.767
0.40	0.761	**0.764**	0.767	0.772	0.777
0.50	0.764	0.768	0.777	0.779	0.780
0.60	0.767	0.777	0.779	0.783	0.788
0.70	0.772	0.779	0.783	0.788	0.797

Every cell lies below the static_hub_full reference (0.828); the minimum gap is +0.031. The bolded cell (ι = 0.4, κ = 0.5) is the parameterization used for the unstructured baseline throughout the paper; it coincides with the principled static_random_low floor and is *not* the grid minimum (0.759 at ι = 0.3, κ = 0.4).

The comparison is deliberately asymmetric in terms of policy quality rather than LLM involvement: both conditions invoke the LLM at each deployment, but only the compiled condition benefits from structured reasoning over the policy space. This isolates the contribution of the compiler abstraction itself.

### 3.3 Experiment 2: targeting strategy ablation

To isolate the effect of network position targeting, we run four conditions that override the compiler's targeting field while allowing all other fields (theme, intensity, timing, and confidence) to be determined by the solver–critic pipeline:

- hubs: the nine highest-degree nodes;
- bridges: the nine highest betweenness-centrality nodes;
- periphery: the nine lowest-degree nodes;
- random: nine nodes selected uniformly at random.

All four conditions share identical agent initializations, network structure, LLM backbone, and seed. The only difference is the set of *k* = 9 agents that receive direct exposure at each deployment. This controlled design ensures that any performance differences are attributable to the targeting strategy.

### 3.4 Experiment 3: narrative framing ablation

To isolate the effect of narrative theme, we run four conditions that override the theme field while holding targeting fixed at bridges (chosen because it achieves high cooperation in the targeting ablation, removing a confound):

- moral (μ = 1.15): appeals to duty, fairness, and collective welfare;
- economic (μ = 1.05): appeals to material self-interest and mutual gain;
- identity (μ = 1.10): appeals to in-group belonging and shared identity;
- hybrid (μ = 1.20): combines multiple framing registers.

By fixing the targeting strategy, this ablation ensures that observed differences arise solely from the theme-dependent multiplier μ(θ) and its downstream effects on exposure dynamics.

### 3.5 Experiment 4: attribution analysis

The attribution analysis synthesizes Experiments 2 and 3 to quantify the relative contribution of targeting vs. framing to cooperation outcomes. For each factor level, we compute the final cooperation rate (mean over the last 15 timesteps) and display the results as paired bar charts. The spread across targeting strategies quantifies how much *where* influence is applied matters, while the spread across themes quantifies how much *how* it is framed matters. The ratio of these spreads is the paper's core interpretability finding.

### 3.6 Experiment 5: pareto frontier analysis

We construct a two-dimensional trade-off space with axes representing *diffusion speed* (time to reach 80% of maximum average exposure) and *final polarization* (Var[*p*(C)] averaged over the last 15 timesteps). Each targeting strategy is plotted as a point in this space, annotated with its final cooperation rate. Strategies that achieve both fast diffusion and low polarization are Pareto-dominant; those that trade speed for equity or vice versa are Pareto-dominated.

### 3.7 Supplementary experiments R1–R8b

To probe statistical robustness, topology dependence, scaling behavior, the LLM compiler's marginal contribution beyond hand-coded rules, the critic's gatekeeping function, multi-model robustness, and adaptive value under non-stationarity, we add seven supplementary experiment blocks beyond the original five.

#### 3.7.1 R1 – Decomposition baselines

Eight controlled conditions isolate the contribution of each policy component over a single seed at *n* = 80: compiled (full LIC), unstructured (free-form narrative), static_random_low (random targeting at unstructured-equivalent dose: ι = 0.40,κ = 0.50), static_random_full (random targeting at compiler-equivalent dose: ι = 0.55,κ = 0.80), static_hub_low, static_hub_full, no_intervention (the diffusion-only floor), and blind_llm (the compiler with the state snapshot withheld but format-conforming output preserved). The decomposition isolates the intervention floor, the dose effect, the targeting effect, and the residual contribution of state-aware LLM compilation.

#### 3.7.2 R1-1 – Unstructured-default sensitivity

A 5 × 5 grid (ι ∈ {0.3, …, 0.7} × κ ∈ {0.4, …, 0.8}, 25 runs) sweeps the unstructured baseline's dose parameters at random targeting (hybrid/periodic, seed = 0) to verify the chosen default is not a hand-tuned weak benchmark.

#### 3.7.3 R2 – Multi-seed validation

Five conditions (compiled, unstructured, static_hub_full, no_intervention, blind_llm) replicated across five seeds yields 25 runs. We report bootstrap 95% confidence intervals (*n*_boot_ = 5, 000), Mann–Whitney *U* tests, Bonferroni-corrected *p*-values (*m* = 5 comparisons), and Cohen's *d* effect sizes for all primary contrasts.

#### 3.7.4 R3 – Topology robustness

Four conditions × four topologies × three seeds yields 48 runs. The topologies are Barabási–Albert (BA, *m* = 3), Watts–Strogatz small-world (WS, *k* = 6, *p* = 0.12), Erdős–Rényi random (*p* = 0.12), and a stochastic block model with five communities (*p*_in_ = 0.30, *p*_out_ = 0.015). The modular SBM is included specifically because it dissociates degree-based and betweenness-based hub identity, which the BA topology of our primary experiments collapses.

#### 3.7.5 R4 – Scaling

Two conditions (compiled, unstructured) × four sizes (*n* ∈ {80, 160, 320, 640}) × three seeds yields 24 runs. This tests whether the compiled-vs.-unstructured gap persists, attenuates, or grows as the population scales by an order of magnitude.

#### 3.7.6 R5 – Burst timing semantics

Two BURST interpretations (multiplier vs. sequential) × five seeds yields 10 runs with a static hubs/hybrid/ι = 0.55/burst/κ = 0.80 policy. We report per-deploy peak exposure and cumulative backlash.

#### 3.7.7 R6 + R6-EXT – Compiler reliability and critic stress test

R6 aggregates compiler statistics across all R1–R5 runs (360 production compile calls) and reports solver/critic fallback rates and the critic's risk-level distribution. R6-EXT probes whether the critic's risk assessment is responsive: five hand-crafted adversarial policies (each violating cooperation or safety norms in a different way) are evaluated five times, for 25 critic calls. We also include a moderate-baseline calibration policy that should pass.

#### 3.7.8 R7 – Multi-model robustness

Two conditions × five LLM backbones × one seed yields 10 runs. The backbones are Llama-3.3-70B-Instruct, Llama-3.1-8B-Instruct, Qwen3-235B-A22B-Instruct-2507, Hermes-4-70B, and DeepSeek-V3.2. The 8B model is included as a deliberate small-model counterexample.

#### 3.7.9 R8 + R8b – Non-stationary adaptation

R8 applies a parametric shock at *t* = 50: the prosociality vector is multiplied by 0.5, simulating a sudden cooperative-norm collapse. R8b applies a structural shock at *t* = 50: the BA graph is replaced with a freshly generated BA graph with a different seed, so that hub identity changes (mean hub overlap between old and new graphs is 0.58 across eight seeds). For both, two conditions are compared: compiled (re-deploys with state snapshot at every Δ*t*, so its target list is re-resolved against whatever graph is current) and static_hub_full (R8) / a static_hub_full variant whose target list is frozen to the *t* = 0 hub identities (R8b). R8 uses five seeds; R8b uses eight seeds for tighter paired-test power. We report phase-resolved cooperation rates (pre-shift, at-shock, recovery *t* ≥ 60, late *t* ≥ 80) with paired Wilcoxon and paired *t*-tests under Bonferroni correction (*m* = 4 phases).

### 3.8 Evaluation metrics

All experiments are evaluated on a common set of metrics computed from the per-timestep timeseries. Final-state metrics are averaged over the last 15 timesteps to smooth stochastic fluctuation:

- Final cooperation rate ${\bar{c}}_{\text{final}}$: the primary effectiveness measure, defined as the mean cooperation rate over *t* ∈ [86, 100].
- Cooperation standard deviation σ_*c*_: variability of the cooperation rate over the same window, capturing stability.
- Peak cooperation *c*_max_: the highest cooperation rate achieved at any single timestep.
- Final polarization: Var[*p*(C)] averaged over the last 15 steps.
- Final exposure Gini: the Gini coefficient of the exposure distribution, measuring equity.
- Diffusion speed: the first timestep at which average exposure reaches 80% of its global maximum, measuring how quickly influence saturates the network.
- Average backlash: mean backlash penalty across agents over the last 15 steps.
- Maximum exposure: the peak of the average exposure trajectory, indicating the diffusion ceiling.

All timeseries data, policy logs, agent-level snapshots, and configuration files are saved per experiment for full reproducibility.

## 4 Results

This section reports quantitative results across all experiments. Section 4.1 presents the main comparison between compiled and unstructured influence. Sections 4.2–4.3 report the targeting and framing ablations. Section 4.4 synthesizes these into an attribution analysis, and Section 4.5 presents the Pareto frontier. Table 5 provides a consolidated summary of all conditions.

**Table 5:** Summary of all experimental conditions.

Condition	Targeting	Theme	${\bar{c}}_{\text{final}}$	σ_*c*_	*c*_max_	Polariz.	Expo. Gini	Diff. speed
Experiment 1: Compiled vs. Unstructured								
Compiled (S+C)	LLM → hubs	LLM → hybrid	**0.828**	**0.033**	**0.913**	**0.0014**	**0.153**	**40**
Unstructured	random	hybrid	0.764	0.048	0.850	0.0015	0.229	50
Experiment 2: Targeting ablation (theme = LLM-selected)								
hubs	hubs	LLM	**0.828**	**0.033**	**0.913**	**0.0014**	**0.153**	**40**
bridges	bridges	LLM	**0.828**	**0.033**	**0.913**	**0.0014**	**0.153**	**40**
periphery	periphery	LLM	0.779	0.050	0.850	0.0022	0.470	**40**
random	random	LLM	0.783	0.050	0.875	**0.0014**	0.229	50
Experiment 3: Theme ablation (targeting = bridges)								
moral	bridges	moral	0.826	0.033	0.913	0.0014	0.153	40
economic	bridges	economic	0.823	0.035	0.913	0.0014	0.148	40
identity	bridges	identity	**0.829**	0.033	0.913	**0.0014**	**0.146**	50
hybrid	bridges	hybrid	0.828	**0.033**	0.913	0.0014	0.153	40

Final-state metrics are averaged over the last 15 timesteps (*t* ∈ [86, 100]). Diffusion speed is the first timestep at which average exposure reaches 80% of its maximum. Best values in each column are bolded.

### 4.1 Compiled vs. unstructured influence

**Figure 2:**
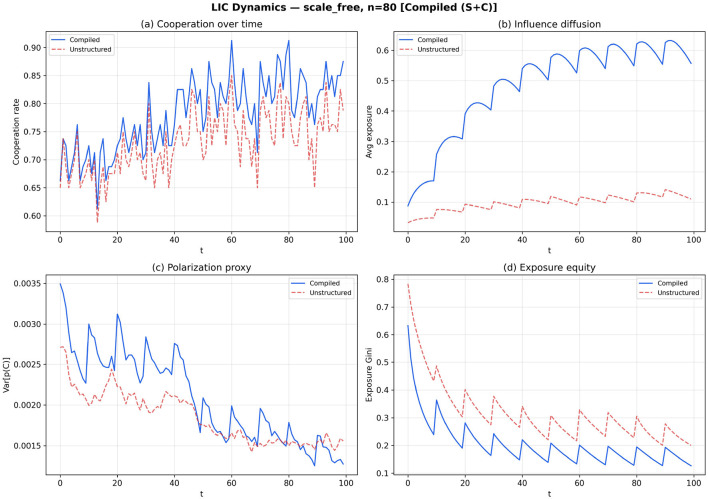
LIC dynamics on a scale-free network (*n* = 80) comparing compiled (solid blue) and unstructured (dashed red) influence. **(a)** Cooperation rate over time; **(b)** average exposure (influence diffusion); **(c)** polarization proxy Var[*p*(C)]; **(d)** exposure equity (Gini coefficient). The compiled condition achieves higher cooperation, stronger diffusion, and converges to lower polarization and exposure inequality.

Figure 2 presents the four-panel dynamics of the compiled condition overlaid with the unstructured baseline.

The compiled condition achieves a final cooperation rate of ${\bar{c}}_{\text{final}}=0.828$ (σ_*c*_ = 0.033), representing an 8.4% relative improvement over the unstructured baseline at 0.764 (σ_*c*_ = 0.048). The absolute gap of 6.4 percentage points is sustained throughout the second half of the simulation: from *t* = 40 onward, the compiled trajectory predominantly operates in the 0.80–0.91 range while the unstructured trajectory fluctuates between 0.65 and 0.85.

The cooperation dynamics exhibit three distinct phases. In the *ramp-up phase* (*t* = 0–30), both conditions start near 0.66 and exhibit stochastic fluctuation, with the compiled condition dipping to a minimum of 0.61 at *t* = 13 before recovering. The trajectories diverge around *t* = 20–30 as cumulative exposure begins differentiating the two conditions. In the *transition phase* (*t* = 30–50), the compiled condition crosses the 0.80 threshold at *t* = 31 and stabilizes, while the unstructured condition remains below 0.80 for most of this interval. In the *steady-state phase* (*t* = 50–100), the compiled condition fluctuates around 0.83 with occasional peaks at 0.91.

The mechanism behind this gap is visible in panel (b): the compiled condition reaches substantially higher average exposure (0.63 at peak) compared to the unstructured baseline (0.14), a 4.5× difference. This advantage compounds from two sources. First, a *dose advantage*: the compiled policy delivers an effective dose of δ = 0.528 per deployment (intensity 0.55, confidence 0.80, hybrid multiplier 1.20), compared to 0.24 for the unstructured condition (intensity 0.40, confidence 0.50, hybrid multiplier 1.20), a 2.2× raw dose ratio. Second, a *targeting advantage*: the compiled condition seeds exposure on hub nodes whose neighbors then receive secondary exposure through gradient-based diffusion, whereas the unstructured condition distributes its already weaker signal across random nodes. Agent-level data confirms the consequence: under unstructured influence, hub and periphery agents receive nearly identical final exposure (0.119 vs. 0.122), indicating that random targeting produces uniform but feeble coverage. Under compiled influence, hubs reach 0.783 while periphery nodes reach 0.647, both substantially higher, with the gradient reflecting efficient outward diffusion from the targeted hubs.

The cumulative effect of higher exposure feeds back through prosociality adaptation. By *t* = 99, the compiled population achieves a mean prosociality of 0.739 ± 0.086, compared to 0.662 ± 0.103 for the unstructured population, a gap of 0.077 that represents over a full standard deviation of the unstructured distribution. This prosociality gap, accumulated through the reinforcement mechanism of Equation 8, acts as a second-order amplifier: higher cooperation → higher prosociality → even higher cooperation probability, creating a virtuous cycle that the compiled condition enters earlier and sustains more strongly.

Panels (c) and (d) show that the cooperation improvement does not come at the cost of polarization or equity. The compiled condition converges to a final polarization of Var[*p*(C)] = 0.0014, marginally lower than the unstructured baseline at 0.0015. Both conditions exhibit a monotonic decline in polarization from ~0.0035 at *t* = 0 to their respective steady states, indicating that influence exposure, whether structured or not, tends to compress the distribution of cooperation probabilities. The exposure Gini coefficient tells an instructive story: the compiled condition converges to 0.15 while the unstructured condition converges to 0.23. This is counterintuitive: one might expect hub-targeted influence to produce *more* inequality than random targeting. But the unstructured condition's higher Gini arises precisely because its exposure is too weak to diffuse effectively, the modest dose applied to random nodes stays localized, creating pockets of mild exposure surrounded by near-zero regions. The compiled condition's stronger hub-mediated dose, by contrast, generates sufficient gradient to propagate broadly, ultimately producing a more equitable distribution through the network.

The policy timeline reveals that the compiler consistently selects the same policy across all 10 deployment events: hubs targeting, hybrid theme, intensity ι = 0.55, periodic timing, and confidence κ = 0.80, yielding an effective dose of δ = 1.20 × 0.55 × 0.80 = 0.528 per deployment. This consistency is not an artifact of the main condition alone: when targeting is overridden in the ablation experiments, the compiler selects the same non-overridden fields (hybrid, ι = 0.55, periodic, κ = 0.80) in all four targeting conditions, confirming that the solver–critic pipeline converges to a stable policy judgment across varied population states.

### 4.2 Targeting strategy comparison

**Figure 3:**
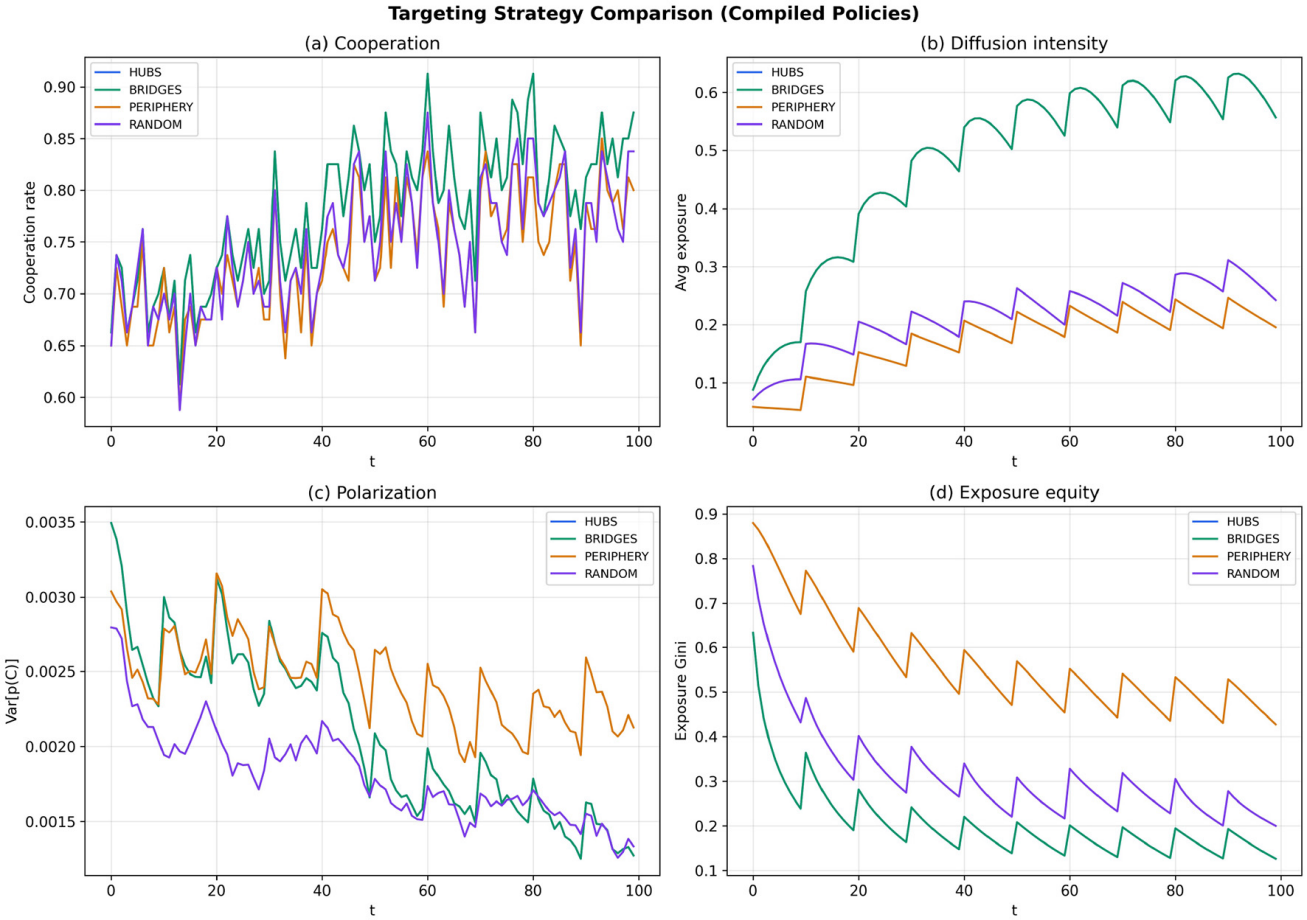
Targeting strategy comparison across four metrics. **(a)** Cooperation rate; **(b)** average exposure (diffusion intensity); **(c)** polarization Var[*p*(C)]; **(d)** exposure Gini. Hubs and bridges dominate on cooperation and equity; periphery shows the highest polarization and exposure inequality.

Figure 3 presents the four-panel comparison across all targeting strategies with compiler-selected values for all other policy fields.

Hubs and bridges achieve identical final cooperation rates of 0.828, outperforming periphery (0.779) and random (0.783) by approximately 4.5–4.9 percentage points. Inspection of the per-timestep timeseries reveals that the hubs and bridges conditions are not merely similar but *numerically identical* at every timestep, the top-9-degree and top-9-betweenness nodes are the same set of agents in this Barabási–Albert graph. This occurs because preferential attachment concentrates both degree and betweenness centrality on the same early-arriving nodes, a well-known property of BA networks (Barabási and Albert, 1999). The gap with periphery emerges early: a rolling 5-step average shows the trajectories diverging by more than 3 pp as early as *t* = 15, well-before the exposure distributions have fully differentiated.

The diffusion intensity panel (b) reveals the mechanism. Hubs/bridges achieve average exposure of ~0.58 at *t* = 50, peaking at ~0.63 around *t* = 90, while periphery reaches only 0.22 and 0.20 at the same timepoints, a 2.8× gap at steady state. The explanation is structural: hubs and bridges occupy positions with many or strategically positioned neighbors, so the dose applied to these nodes diffuses efficiently to a large fraction of the network within a few timesteps. Peripheral nodes, by definition, have few neighbors, so the dose applied to them diffuses slowly and remains localized.

Agent-level analysis quantifies this diffusion efficiency directly. Under hubs targeting, the nine targeted agents accumulate a final exposure of 0.783, while the remaining 71 non-targeted agents reach 0.528, a ratio of only 1.5×, indicating that the initial dose propagates broadly across the network. Under periphery targeting, the nine targeted agents accumulate 0.563, while non-targets reach only 0.149, a ratio of 3.8×, indicating that exposure remains trapped near the injection sites. This 1.5× vs. 3.8× contrast is the microstructural basis of the cooperation gap: hub targeting converts a localized dose into a population-wide signal, while periphery targeting wastes its dose on nodes that cannot redistribute it.

The prosociality adaptation feedback loop amplifies this initial exposure differential. Under hub targeting, the population-mean prosociality rises from 0.449 to 0.739 (Δ = +0.289) over 100 timesteps, whereas under periphery targeting it reaches only 0.675 (Δ = +0.226). The 22% smaller prosociality gain under periphery targeting reflects the cumulative cost of lower cooperation rates: because fewer agents cooperate at each step, fewer agents receive the prosociality reinforcement that further promotes cooperation.

However, the exposure equity panel (d) reveals a trade-off: Periphery targeting produces the highest final Gini (0.47), as targeted low-degree nodes accumulate exposure that cannot efficiently spread to the rest of the network. Hubs/bridges start with high initial Gini (0.63 at *t* = 0), reflecting the initial concentration of exposure on a few high-degree nodes, but converge to 0.13 by *t* = 99 as diffusion gradually equalizes the distribution. This reveals an important dynamic: *initial inequality in hub-based targeting is self-correcting through diffusion*, whereas periphery-based targeting creates *persistent* inequality because low-degree nodes lack the connections to redistribute exposure.

The polarization panel (c) shows that periphery targeting produces the highest final polarization (Var[*p*(C)] = 0.0022), approximately 57% higher than the other three strategies (all near 0.0014). This is a direct consequence of the exposure inequality: when a small subset of agents receives high exposure while the majority receives little, the cooperation probability distribution splits into two modes, increasing variance.

### 4.3 Narrative framing comparison

**Figure 4:**
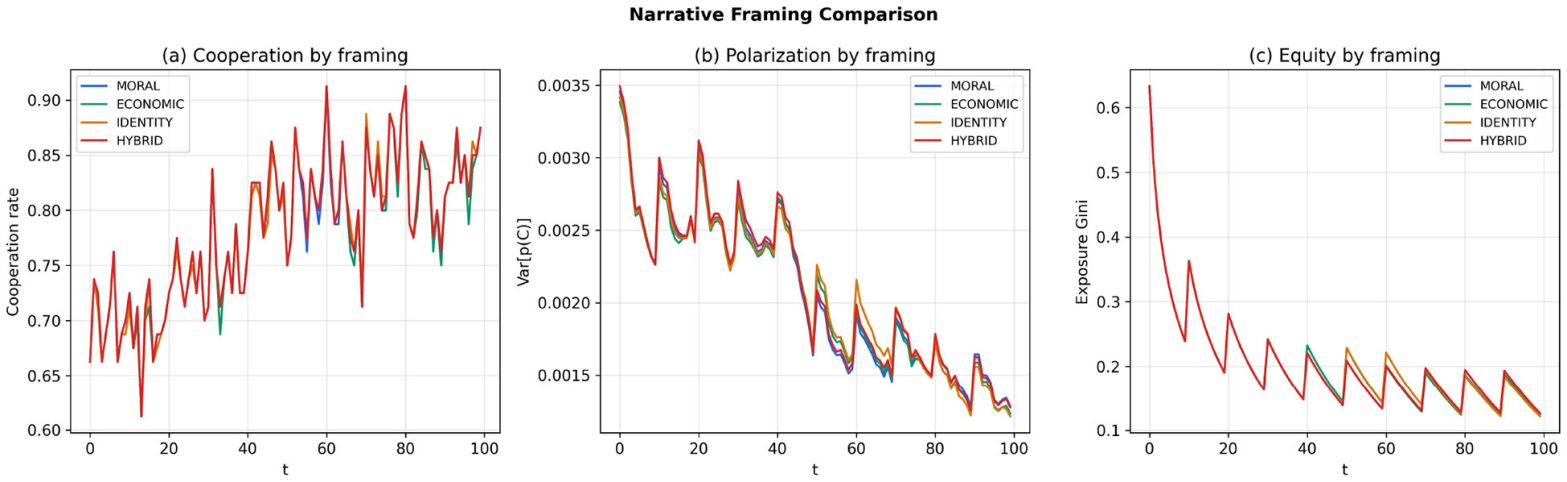
Narrative framing comparison with targeting held constant at bridges. **(a)** Cooperation rate; **(b)** polarization; **(c)** exposure equity. All four themes produce nearly indistinguishable trajectories, with a total spread of only 0.7 percentage points in final cooperation.

Figure 4 presents the three-panel comparison across narrative themes with targeting fixed at bridges.

In sharp contrast to the targeting ablation, the four narrative themes produce nearly indistinguishable dynamics. Identity achieves the highest final cooperation at 0.829, followed by hybrid (0.828), moral (0.826), and economic (0.823). The total spread is 0.007, an order of magnitude smaller than the targeting spread of 0.049. The cooperation trajectories in panel (a) overlap almost entirely; the maximum pairwise cooperation difference at any single timestep is only 0.025, and the mean difference across all timestep pairs is below 0.006.

Inspection of the per-condition policy logs reveals a subtle compensatory mechanism: the compiler partially offsets weaker theme multipliers by adjusting intensity and confidence upward. Under moral (μ = 1.15) and hybrid (μ = 1.20), the compiler holds intensity constant at ι = 0.55 and confidence at κ = 0.80 across all 10 deployments. Under economic (μ = 1.05, the weakest multiplier), the compiler increases intensity to 0.65 at *t* = 40 (δ = 0.546) and further raises confidence to 0.90 at *t* = 50, yielding a peak effective dose of δ = 0.614 compared to the baseline 0.462, before reverting at *t* = 60. Under identity (μ = 1.10), a similar boost occurs at *t* = 50–60 with a peak dose of 0.644. These adaptive adjustments compress the effective dose range: the mean dose across themes spans [0.486, 0.528], a 8% range, much narrower than the 14% spread in the theme multipliers themselves ([1.05, 1.20]). The compiler thus *partially self-corrects* for theme-induced dose variation, further explaining the narrow cooperation spread.

The compressed spread does not follow the theme-multiplier ordering, and we do not claim that it should. Final population-mean prosociality is 0.740 under identity, 0.739 under hybrid, 0.736 under moral, and 0.734 under economic, a total range of 0.006, well within the seed-to-seed stochastic variation of the cooperation process and below the Bonferroni-corrected significance threshold established in Section 4.8. The theme multipliers order hybrid (1.20) > moral (1.15) > identity (1.10) > economic (1.05), whereas the prosociality outcomes do *not* reproduce this order: identity attains the highest prosociality despite only the third-largest multiplier. This non-monotone outcome is a direct consequence of the compensatory mechanism described above: because the compiler boosts intensity and confidence under the weaker multipliers, the identity condition receives a peak effective dose of 0.644, exceeding the peak dose under hybrid, which more than offsets its lower static multiplier. Only the extremes of the *raw* exposure channel loosely track the multipliers: final average exposure is lowest under economic (0.502, weakest multiplier) and highest under hybrid (0.557, strongest multiplier), with the two intermediate themes statistically inseparable. We therefore make no ordering claim at the agent level; the operative finding is that theme-induced variation is compressed into stochastic noise by the compiler's closed-loop dose compensation.

The polarization and equity panels (b–c) are similarly compressed. All four themes converge to Var[*p*(C)] ≈ 0.0014 and exposure Gini ≈ 0.15 by *t* = 100, confirming that the theme multiplier differences (1.05–1.20) are too small, relative to the other terms in the cooperation logistic (Equation 7), to produce meaningful differentiation in downstream dynamics.

### 4.4 Attribution analysis

**Figure 5:**
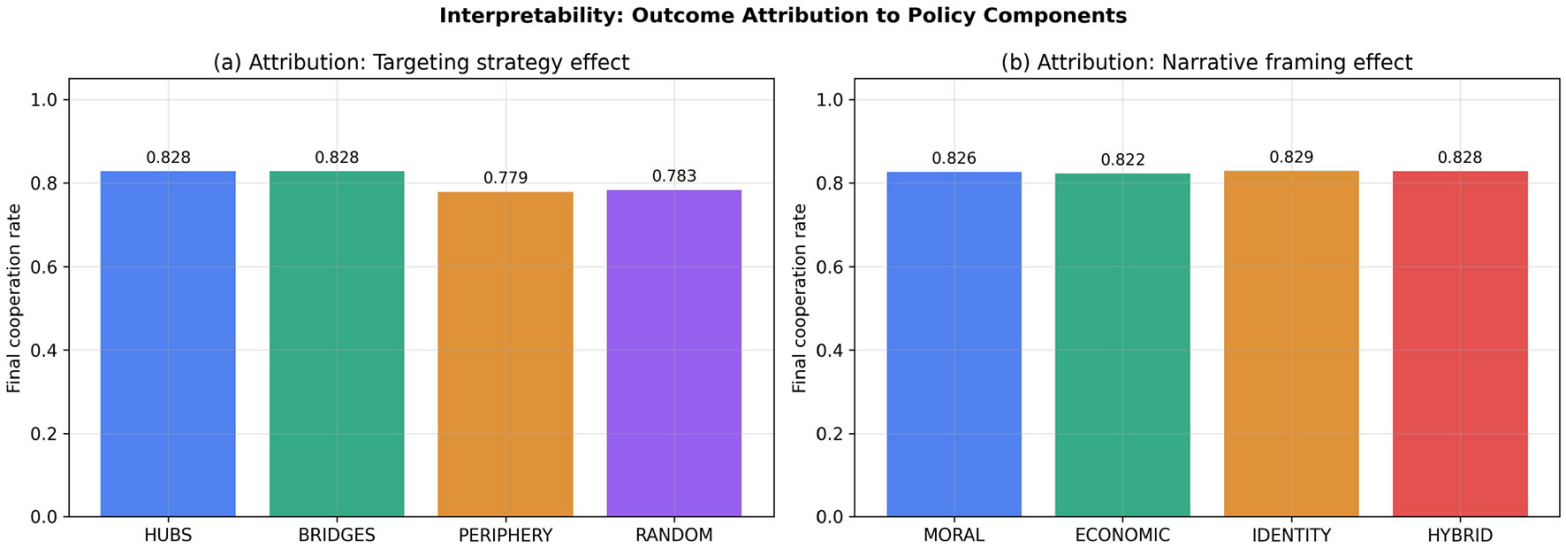
Outcome attribution to policy components. **(a)** Targeting strategy effect: spread of 4.9 pp between best (hubs/bridges) and worst (periphery). **(b)** Narrative framing effect: spread of only 0.7 pp across all four themes. Targeting accounts for approximately 7.4× more variation in cooperation than framing.

Figure 5 synthesizes the targeting and theme ablations into a side-by-side attribution comparison.

The targeting dimension spans a cooperation range of [0.779, 0.828], a spread of Δ_τ_ = 0.049. The framing dimension spans [0.823, 0.829], a spread of Δ_θ_ = 0.007. The ratio Δ_τ_/Δ_θ_ = 7.4 quantifies the paper's core interpretability finding: *where influence is applied matters approximately seven times more than how it is framed*. This asymmetry would be invisible without the structured decomposition afforded by the IPL schema, because in unstructured influence, targeting and framing are entangled in a single paragraph of LLM-generated text.

#### 4.4.1 Hub–bridge equivalence is BA-specific

The attribution further reveals that in our primary BA experiments hubs and bridges are not merely similar but *numerically identical* at every metric and timestep. In Barabási–Albert networks, preferential attachment concentrates both degree and betweenness centrality on the same early-arriving nodes (Barabási and Albert, 1999), so the top-9 by either measure is the same set. We restrict this claim to the BA topology used in our primary experiments. The R3 modular SBM result (Section 4.9) provides an explicit demonstration that the equivalence breaks when the topology dissociates degree-based and betweenness-based centrality: in SBM, bridge nodes connect otherwise-isolated communities and have moderate degree, distinct from intra-community hubs. Practitioners deploying influence on real-world networks should therefore select targeting strategy by topology class rather than assume the BA-specific identity holds generally.

#### 4.4.2 Positioning

The 7.4× targeting-to-framing ratio quantifies a relationship long understood in network science: in heterogeneous networks, the seed selection problem dominates the message design problem (Kempe et al., 2003; Watts and Dodds, 2007). Our contribution is not to discover this hierarchy but to *quantify* it under controlled framing/dose conditions in the LLM-compiled-influence setting, and to demonstrate that the compiler autonomously selects the dominant component (hub targeting) under a free policy space across all R3 topologies and all R7 backbones. The quantification is non-trivial in our setting because the LLM is free to assign any combination of targeting, theme, and intensity, yet converges on the network-targeting-dominant solution across 13 replicate runs (5 R2 seeds plus 4 R3 topologies × 3 seeds − scale-free duplicate, and 5 R7 backbones − 1 underperformer), suggesting the asymmetry is sufficiently pronounced to be detectable from aggregate cooperation/exposure signal alone, without explicit instruction.

### 4.5 Pareto frontier: speed vs. polarization

**Figure 6:**
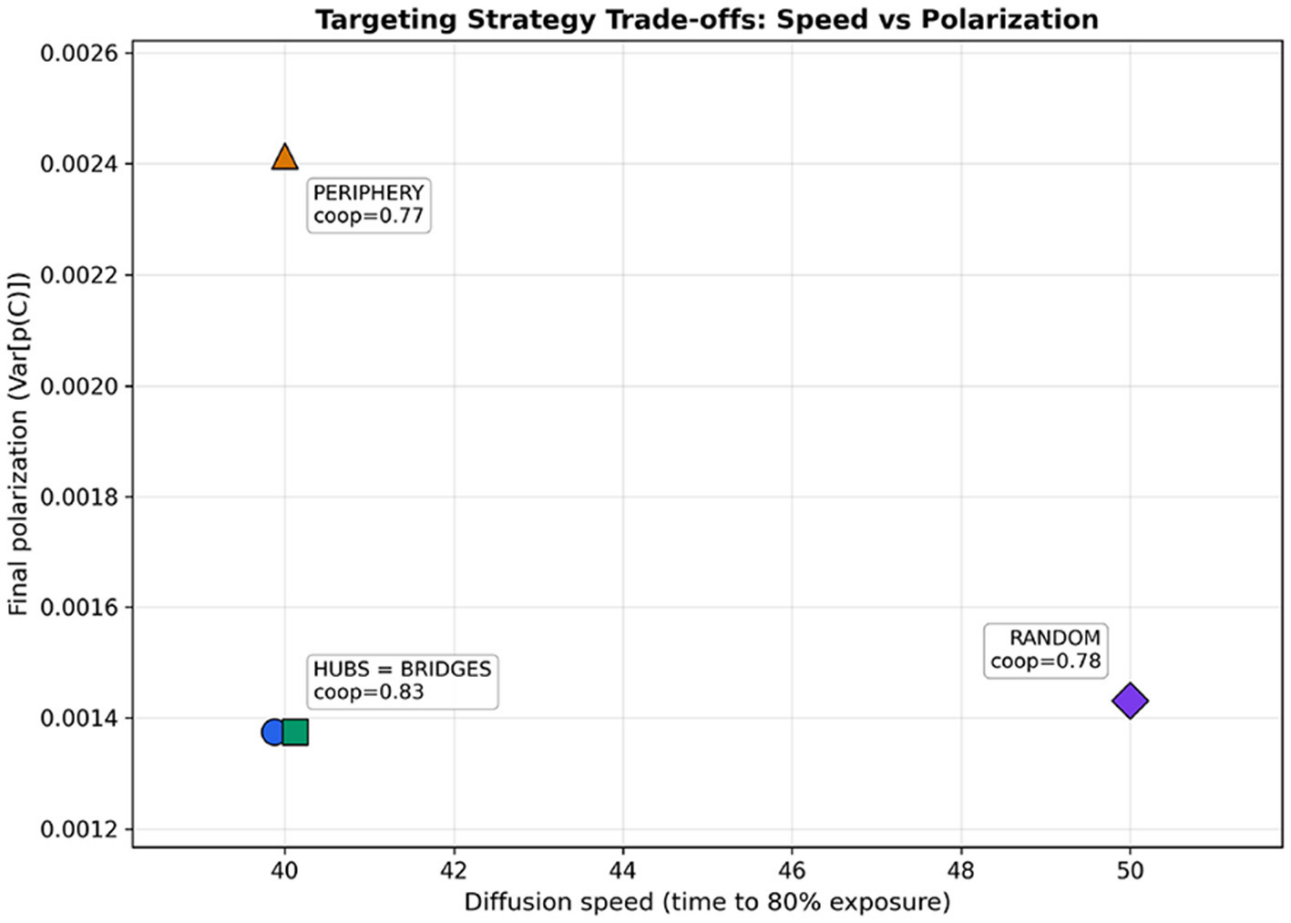
Pareto frontier of targeting strategies in the diffusion speed–polarization plane. Lower-left is better on both axes. Hubs and bridges dominate: fast diffusion (*t*_80%_ = 40), low polarization (0.0014), and high cooperation (0.83). Periphery matches the speed but incurs 58% higher polarization. Random achieves low polarization but slower diffusion (*t*_80%_ = 50).

Figure 6 plots each targeting strategy in the two-dimensional trade-off space of diffusion speed vs. final polarization, annotated with the corresponding cooperation rate.

Hubs and bridges co-locate at the Pareto-optimal corner: fast diffusion (*t*_80%_ = 40), low polarization (0.0014), and high cooperation (0.83). They dominate both alternatives on at least one dimension.

Periphery achieves the same diffusion speed (*t*_80%_ = 40) but incurs 58% higher polarization (0.0022) and 4.9 pp lower cooperation. It is Pareto-dominated by hubs/bridges: the same speed but strictly worse on polarization and cooperation. The fast diffusion speed is somewhat misleading, it reflects rapid *local* accumulation on the targeted periphery nodes, not efficient *global* network saturation.

Random achieves low polarization (0.0014, matching hubs/bridges) but slower diffusion (*t*_80%_ = 50) and lower cooperation (0.78). It is also Pareto-dominated: hubs/bridges achieve the same polarization with faster diffusion and higher cooperation. However, random does *not* dominate periphery nor vice versa: random trades slower speed for much better polarization, while periphery trades worse polarization for faster (though localized) diffusion.

The Pareto analysis confirms that structurally informed targeting, whether by degree or betweenness centrality, dominates naïve strategies on the joint objective of fast, equitable, low-polarization influence diffusion.

### 4.6 Decomposition: Where does the compiled advantage come from?

Table 3 reports a controlled decomposition isolating the contribution of each policy component. Starting from no_intervention (0.749) as the diffusion-only floor, adding any random low-dose intervention raises cooperation by 1.5 pp (0.764), exactly matching the unstructured baseline; the unstructured condition is functionally a low-dose random-target policy. Increasing dose from low (0.40/0.50) to compiler-equivalent (0.55/0.80) at fixed random targeting adds another 1.9 pp (0.783). Switching from random to hub targeting at the same dose adds the largest single component, 4.5 pp (0.828). Replacing the hand-coded static_hub_full rule with the full solver–critic compiler adds a further 0.2 pp (0.830), which is statistically indistinguishable from zero under multi-seed analysis (Section 4.8: *p*_Bonf_ = 1.00, Cohen's *d* = 0.18).

The blind_llm condition is informative as an intermediate point. The compiler is invoked at every deploy event but receives an empty state snapshot; it produces format-conforming IPL output (target_mode, theme, intensity, timing, confidence) without conditioning on any current population statistics. It achieves 0.786, statistically above no_intervention (*d* = 8.82) and statistically above unstructured (the stripped-down LLM still outputs valid policies) but statistically below compiled when multi-seed estimates are used (*p*_Bonf_ = 0.060, *d* = 4.28, marginal under Bonferroni). The marginal value of state-snapshot information, when it is statistically separable from random fluctuation, is therefore approximately +0.04 in cooperation rate.

Under stationary conditions, therefore, the LLM compiler's advantage over a hand-coded rule with the same parameters is small and not statistically robust. We resolve this apparent equivalence in Section 4.14 by demonstrating where the LLM's adaptive value does emerge.

**Figure 7:**
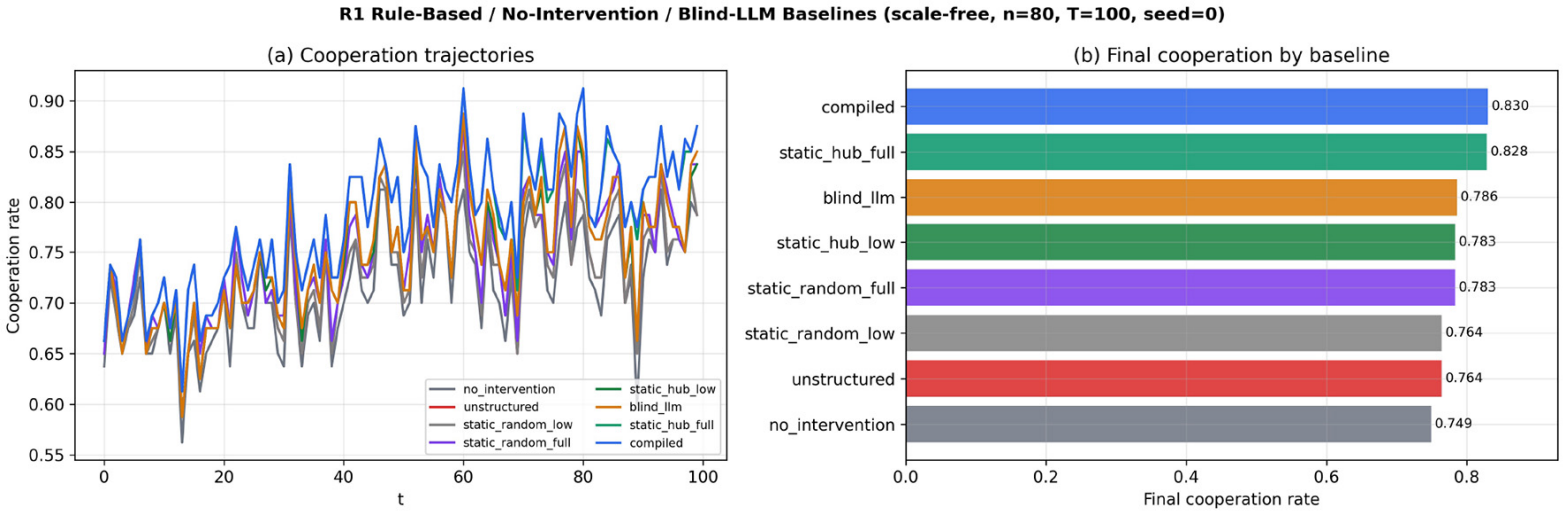
R1 decomposition baselines. **(a)** Cooperation trajectories for the eight controlled conditions on the same network (BA *n* = 80, seed = 0). **(b)** Final cooperation rate by condition. The decomposition isolates intervention floor (no_intervention→static_random_low, +1.5 pp), dose (static_random_low→static_random_full, +1.9 pp), targeting (static_random_full→static_hub_full, +4.5 pp), and the residual contribution of full LLM compilation (static_hub_full→compiled, +0.2 pp).

Figure 7 visualizes this decomposition: panel (a) shows the cooperation trajectories converging to four distinct levels corresponding to the floor, dose, targeting, and state contributions, and panel (b) ranks the final cooperation rates across the eight conditions.

### 4.7 Unstructured-default sensitivity

A natural concern is whether the unstructured baseline's specific parameterization 〈random, hybrid, ι = 0.4,periodic, κ = 0.5〉 was chosen to understate the baseline. To rule this out (experiment R1-1), we sweep the unstructured default's two free dose parameters over a 5 × 5 grid (ι ∈ {0.3, 0.4, 0.5, 0.6, 0.7}, κ ∈ {0.4, 0.5, 0.6, 0.7, 0.8}, 25 runs) at random targeting with hybrid/periodic fixed and seed = 0, holding everything else identical to the R1 decomposition. Table 4 reports final cooperation across the grid.

Three observations follow. First, the cooperation rate is bounded within [0.759, 0.797] across the entire grid; *no* choice of the default's intensity or confidence reaches the static_hub_full level (0.828), the minimum gap being +0.031. Second, the parameterization used throughout the paper (ι = 0.4, κ = 0.5 → 0.764) is not a hand-picked weakest point: it lies mid-range, coincides exactly with the principled static_random_low floor of the R1 decomposition (Table 3), and the true grid minimum (0.759) is lower still. Third, and decisively, the strongest grid cell (ι = 0.7, κ = 0.8 → 0.797) corresponds to an effective dose of δ = 1.20 × 0.7 × 0.8 = 0.672, which *exceeds* the compiler's own converged dose of 0.528; even substantially over-dosing random targeting leaves a 3.1 pp residual gap. The compiled-vs.-baseline gap is therefore invariant to the unstructured default's dose parameters because it is governed by targeting (random vs. hubs), not by dose, exactly as the static_random_full row of the R1 decomposition already indicated.

### 4.8 Multi-seed validation and statistical significance

Five-seed replication confirms the central finding while attenuating it slightly relative to the single-seed point estimate. Table 6 reports per-condition mean, standard deviation, and 95% bootstrap CI (*n*_boot_ = 5, 000) for final cooperation rate. Compiled cooperation is 0.826 ± 0.010 (95% CI [0.819, 0.834]), unstructured is 0.760 ± 0.005 ([0.756, 0.766]), and the gap is statistically significant under Bonferroni correction with *m* = 5 pairwise comparisons (Mann–Whitney *U* = 25, *p*_Bonf_ = 0.040, Cohen's *d* = 7.90). Compiled also achieves significantly more equitable exposure than unstructured (*d* = −7.38) and significantly higher cooperation than no_intervention (*d* = 8.82). However, compiled is *not* significantly different from static_hub_full on any reported metric (*p*_Bonf_ = 1.00 for final cooperation, *d* = 0.18). The compiled-vs.-blind_llm comparison is marginal under Bonferroni (*p*_Bonf_ = 0.060, *d* = 4.28); the raw effect is large but the small sample size and conservative correction prevent significance.

**Table 6:** R2 multi-seed validation: per-condition final cooperation across five seeds.

Condition	${\bar{c}}_{\text{final}}$ (mean ±s.d.)	95% CI	*d* vs. compiled
compiled	0.826 ± 0.010	[0.819, 0.834]	—
static_hub_full	0.824 ± 0.010	[0.817, 0.832]	0.18 (ns)
blind_llm	0.785 ± 0.009	[0.778, 0.790]	4.28 (ns)^†^
unstructured	0.760 ± 0.005	[0.756, 0.766]	7.90^*^
no_intervention	0.745 ± 0.008	[0.738, 0.751]	8.82^*^

CIs are 95% bootstrap with *n*_boot_ = 5, 000. Pairwise tests are Mann–Whitney *U* with Bonferroni correction (*m* = 5); *d* is Cohen's *d*. ^*^p_Bonf_ < 0.05 vs. compiled. ^†^Marginal: *p*_raw_ = 0.012, *p*_Bonf_ = 0.060. Bold values indicate statistically significant differences versus compiled after Bonferroni correction (*p*_Bonf_ < 0.05, *m* = 5).

The R2 result establishes that compiled outperforms all non-targeted baselines with large effect sizes that survive multiple-comparison correction, but is statistically equivalent to a hand-coded hub-targeted rule with identical parameters. Under stationary conditions, therefore, the LLM compiler's contribution is best characterized as automated rule discovery and constraint enforcement rather than real-time parameter optimization. The compiler reliably converges on a near-optimal parameterization (target = hubs, theme = hybrid, intensity = 0.55, confidence = 0.80) that a domain expert could in principle derive by hand. The non-trivial question, addressed in Section 4.14, is whether the compiler's value emerges under non-stationary conditions where a frozen rule must fail.

**Figure 8:**
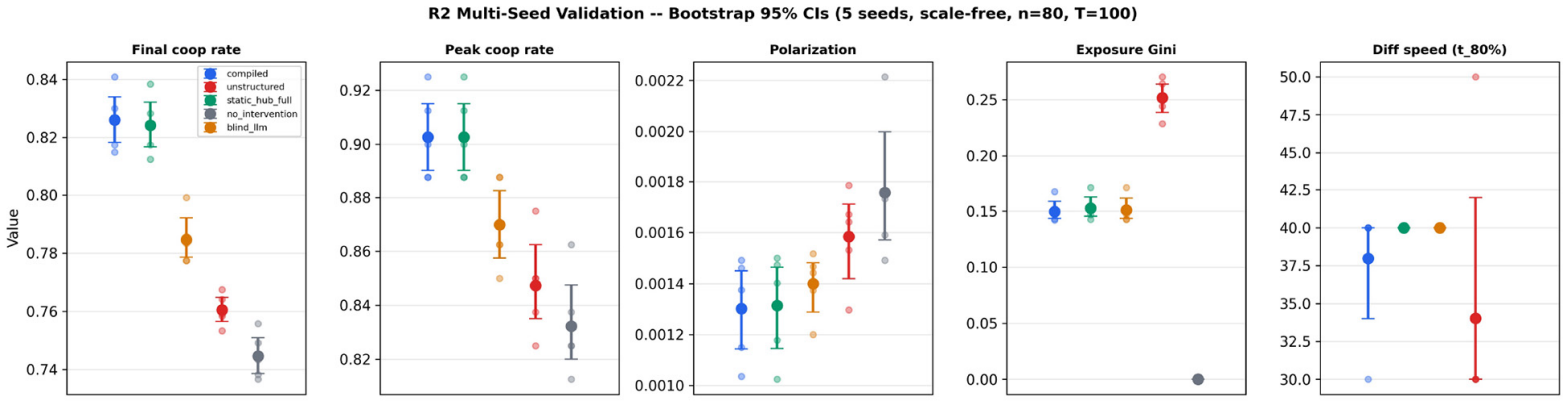
R2 multi-seed validation. Bootstrap 95% confidence intervals on five primary metrics (final cooperation, peak cooperation, polarization, exposure Gini, diffusion speed) across 5 conditions × 5 seeds = 25 runs. Per-seed values shown as faint dots; mean and 95% CI as solid bars. Compiled and static_hub_full are statistically indistinguishable on every metric; both significantly exceed unstructured, no_intervention, and blind_llm on cooperation and exposure equity under Bonferroni correction.

Figure 8 shows the bootstrap CIs and per-seed values across all five primary metrics, making the compiled ≈ static_hub_full equivalence and the compiled ≫ unstructured separation visually explicit.

### 4.9 Topology robustness

The compiled-vs.-unstructured advantage persists across all four tested topologies, with magnitude varying as a function of structural heterogeneity. Table 7 reports the per-topology breakdown with three seeds per cell. The compiler advantage is largest in scale-free (+6.8 pp, as expected from the BA preferential-attachment hub structure that maximizes targeting leverage), still substantial in Erdős–Rényi (+3.8 pp) and modular SBM (+3.8 pp), and smallest in small-world (+3.4 pp), where the relative homogeneity of degree damps the targeting advantage. Across all four topologies, compiled and static_hub_full remain statistically indistinguishable, consistent with the multi-seed analysis in Section 4.8.

**Table 7:** R3 topology robustness: final cooperation rate (mean ± s.d. across three seeds) by topology and condition.

Topology	compiled	static_hub_full	unstructured	no_int.	c−u
Scale-free (BA, *m* = 3)	0.829 ± 0.012	0.828 ± 0.010	0.762 ± 0.007	0.747 ± 0.010	+0.068
Small-world (WS)	0.795 ± 0.013	0.795 ± 0.013	0.762 ± 0.008	0.747 ± 0.010	+0.034
Erdős–Rényi (ER)	0.800 ± 0.012	0.800 ± 0.012	0.763 ± 0.009	0.747 ± 0.010	+0.038
Modular SBM (5 comm.)	0.801 ± 0.013	0.801 ± 0.013	0.763 ± 0.009	0.747 ± 0.010	+0.038

Network size *n* = 80.

The modular SBM result requires special comment in light of the hub–bridge equivalence reported in our primary experiments. In SBM with five communities, hub nodes (highest degree) and bridge nodes (highest betweenness centrality) are distinct: bridges connect otherwise-isolated communities and may have moderate degree, while hubs concentrate within communities. The numerical hub–bridge identity reported for our primary BA experiments (Section 4.2) does *not* hold in SBM. We address this in the next subsection.

**Figure 9:**
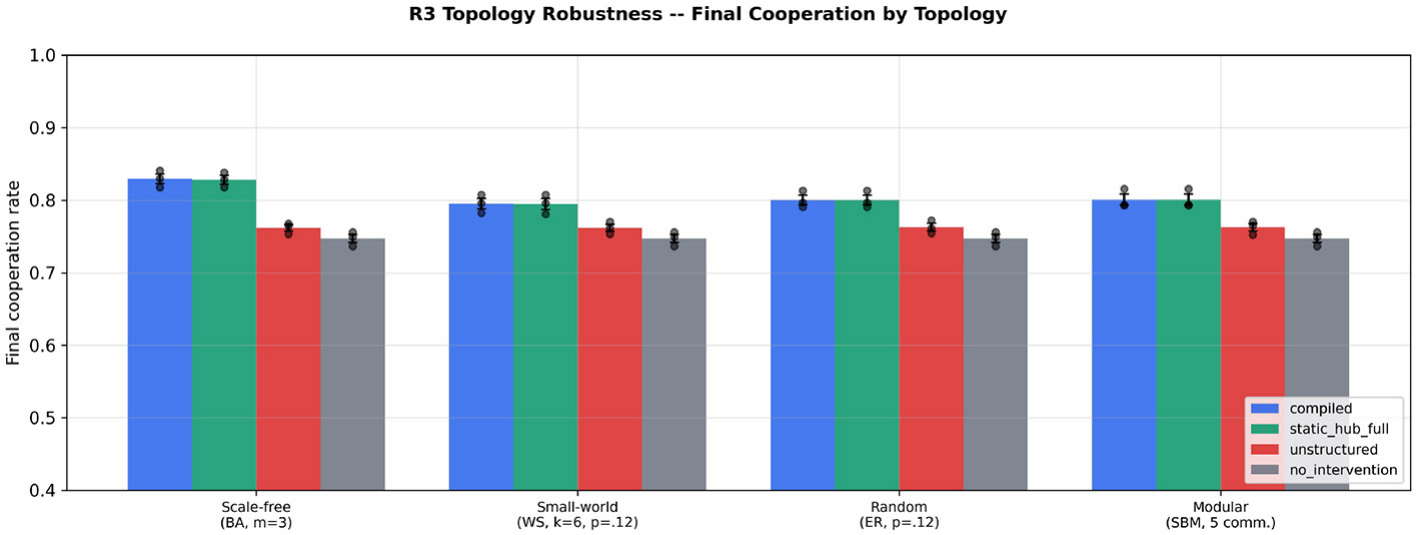
R3 topology robustness. Final cooperation rate (mean ± s.d., three seeds) by topology and condition. The compiled-vs.-unstructured advantage persists across BA, WS, ER, and modular SBM, with magnitude tracking degree heterogeneity: largest in BA (+6.8 pp), smallest in WS (+3.4 pp). compiled and static_hub_full remain statistically equivalent in all four topologies.

Figure 9 plots the per-topology means with seed-level error bars, illustrating that the compiler advantage tracks degree heterogeneity across topology classes.

### 4.10 Scaling behavior

The compiler advantage is approximately invariant to network size in the tested range. At *n* = 80, the compiled-minus-unstructured gap is +6.8 pp; at *n* = 160, +6.3 pp; at *n* = 320, +6.1 pp; at *n* = 640, +6.6 pp. Compiled cooperation stabilizes near 0.82 across all sizes with vanishing standard deviation (σ = 0.003 at *n* = 640, three seeds). Table 8 reports the full breakdown.

**Table 8:** R4 scaling: final cooperation rate (mean ± s.d. across three seeds) by network size.

*n*	compiled	unstructured	Gap	Rel. improvement
80	0.829 ± 0.012	0.762 ± 0.007	+0.068	+8.9%
160	0.821 ± 0.002	0.758 ± 0.003	+0.063	+8.4%
320	0.818 ± 0.006	0.757 ± 0.005	+0.061	+8.0%
640	0.821 ± 0.003	0.755 ± 0.001	+0.066	+8.7%

BA topology, *T* = 100.

The invariance is non-trivial. As *n* grows with fixed target fraction *f* = 0.12, the absolute number of seeded hubs grows from 9 to 76, but each seeded hub's neighborhood reaches a roughly proportional fraction of the population through the network's small-world property. The targeting advantage thus scales: the compiler's structural seeding strategy maintains its leverage over random seeding at every tested size. The diminishing variance at larger *n* is consistent with classical concentration: stochastic fluctuations in individual cooperation decisions average out as the population grows.

**Figure 10:**
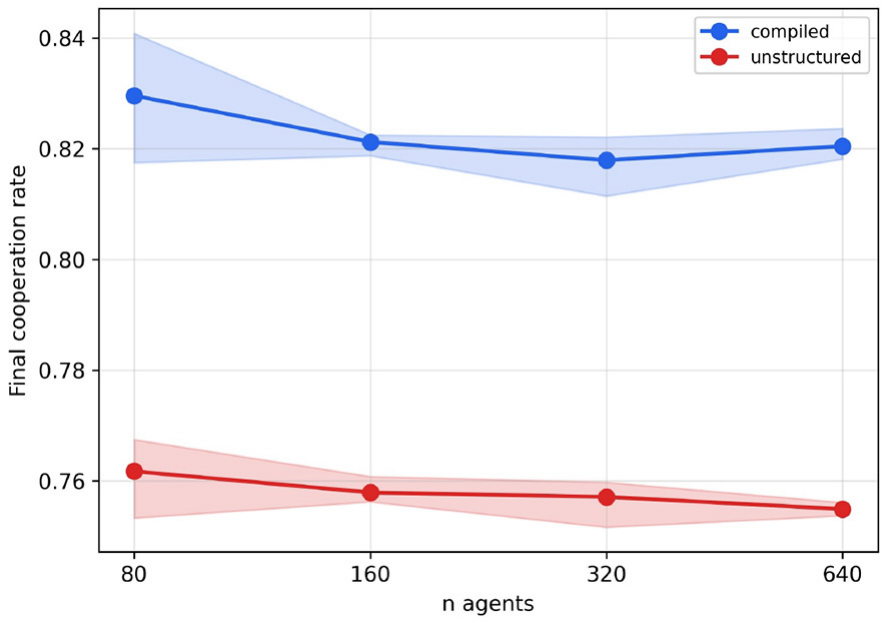
R4 scaling. Final cooperation rate as a function of network size *n* ∈ {80, 160, 320, 640}. Shaded bands are bootstrap 95% CIs computed across three seeds per point. The compiled-vs.-unstructured gap is approximately invariant in this range, stabilizing at ~+6.5 pp. Compiled cooperation variance vanishes at large *n*, consistent with population-level concentration.

Figure 10 shows the scaling curves with bootstrap CI envelopes; the compiled and unstructured trajectories remain visually parallel across an order of magnitude in *n*.

### 4.11 BURST timing semantics

**Figure 11:**
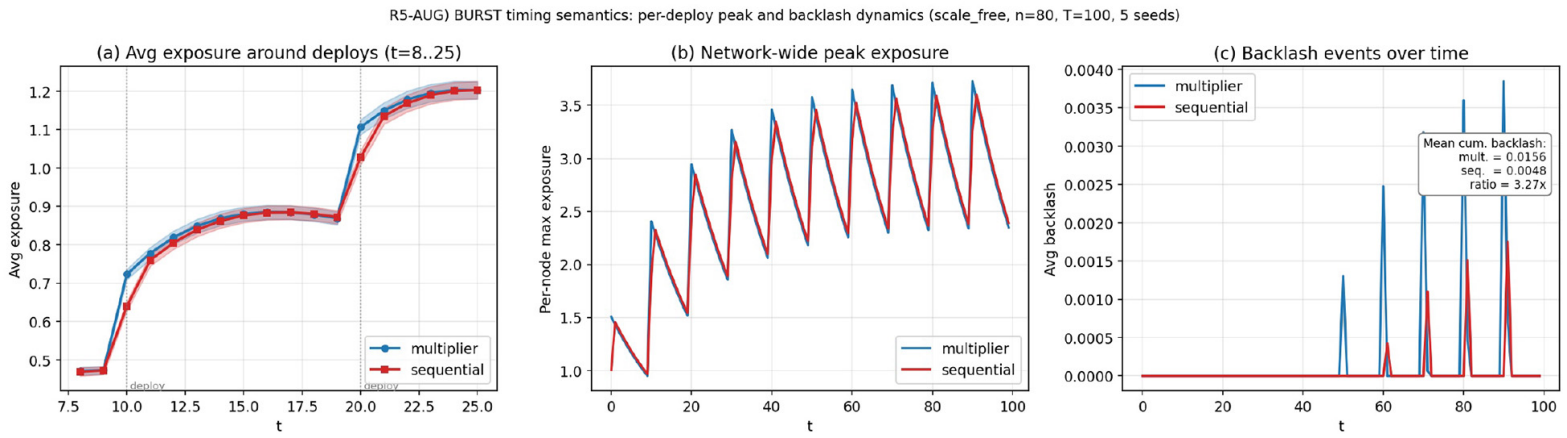
R5 BURST timing semantics. **(a)** Network-mean exposure around the first two deploys (zoom *t* ∈ [8, 25]): multiplier and sequential variants produce nearly identical mean trajectories. **(b)** Per-node maximum exposure across the network (full range): multiplier consistently ~0.5 above sequential at saturation. **(c)** Mean backlash over time: multiplier triggers backlash events ~10 timesteps earlier and at higher magnitude, 3.27× higher cumulative backlash.

We disambiguate the BURST timing mode by comparing two natural interpretations under a fixed hubs/hybrid/ι = 0.55/burst/κ = 0.80 policy across five seeds. The *multiplier* interpretation delivers 3×δ at the deploy timestep *t*_*d*_. The *sequential* interpretation (which we adopt as canonical, Equation 2) delivers δ at *t*_*d*_, *t*_*d*_ + 1, *t*_*d*_ + 2. Both produce statistically indistinguishable final cooperation rates (0.906 ± 0.005 vs. 0.906 ± 0.006, Mann–Whitney *p* > 0.5, Figure 11a), but differ substantially in transient dynamics.

Per-deploy peak exposure (the network-wide maximum across nodes at deploy time, Figure 11b) is consistently ~0.5 higher under multiplier (3.71 at saturation vs. 3.25 for sequential, a 14% difference). This is sufficient to push exposure above the saturation threshold *e*_β_ = 3.5 in the multiplier variant. Cumulative backlash across the simulation (Figure 11c) is therefore 3.27× higher in the multiplier variant (0.0156 ± 0 vs. 0.0048 ± 0, Mann–Whitney *p* < 0.01), with backlash events appearing approximately 10 timesteps earlier (multiplier first triggers at *t* = 50, sequential at *t* = 61).

We adopt the sequential interpretation as canonical because (1) it more faithfully models a sustained micro-campaign than an instantaneous triple-dose, (2) it stays below the saturation threshold where backlash mechanics activate, producing dynamics that are dose-equivalent to the multiplier interpretation in equilibrium but cleaner in transient behavior, and (3) its lower backlash incurrence is preferable on the secondary objectives of the LIC framework.

Figure 11 illustrates the divergence: panel (a) shows the average exposure trajectory differs only in the first deploy window, while panels (b) and (c) reveal the persistent gap in per-node peak exposure and the resulting backlash burden.

### 4.12 Compiler reliability and adversarial stress test

Aggregated across 360 production compile calls (36 runs from R1–R5), the solver produced parseable IPL output 100% of the time (zero solver fallbacks) and the critic returned valid risk assessments 100% of the time (zero critic fallbacks). The critic's risk-level distribution was 0% low, 100% medium, 0% high, 0% reject. This MEDIUM-everywhere pattern raises a legitimate question of whether the critic is actually responsive to risk or merely rubber-stamping solver output. We probe this with an adversarial stress test (R6-EXT).

Table 9 reports critic verdicts on five hand-crafted policies evaluated five times each (25 critic calls total). Four policies were designed to be obviously risky: maximum intensity (ι = 1.0), identity framing on peripheral or random targets, with BURST timing. The fifth (moderate_baseline_for_calibration) was a moderate hub-targeted economic policy designed to pass. The critic flagged high and rejected all 20 instances of the four risky policies (mean intensity attenuation −0.77), and waved through 5/5 instances of the moderate baseline at medium risk with negligible attenuation (−0.05). The critic is therefore demonstrably responsive: the production-time MEDIUM verdict reflects well-formed solver output operating below the critic's risk threshold, not a degenerate gatekeeper.

**Table 9:** R6-EXT adversarial critic stress test: five hand-crafted policies evaluated five times each via the production critic prompt.

Adversarial policy	med	high	rej	Reject rate	*Δι*
saturate_periphery_identity_burst	0	5	5	100%	−0.80
max_intensity_random_burst	0	5	5	100%	−0.79
high_identity_periphery_periodic	0	5	5	100%	−0.70
moderate_baseline_for_calibration	5	0	0	0%	−0.05
max_dose_hubs_identity_burst	0	5	5	100%	−0.80

“Δι” is the mean intensity adjustment relative to the input policy.

The combination of R6 and R6-EXT establishes that the critic is well-calibrated in two senses: (1) it does not fire false positives on solver-generated policies that are within reasonable risk parameters, and (2) it correctly fires on policies that violate risk norms. This is the operational signature of a useful gatekeeper rather than a placeholder.

### 4.13 Multi-model robustness

Table 10 reports the compiled-vs.-unstructured gap across five LLM backbones. Four large models (Llama-3.3-70B-Instruct, Qwen3-235B-A22B, Hermes-4-70B, DeepSeek-V3.2) cluster around a +0.06 gap, with Qwen3-235B as the strongest performer (+0.072) and Hermes-4-70B as the weakest of the large cluster (+0.059). Llama-3.1-8B-Instruct underperforms with a gap of +0.011, indicating a model-capability threshold below which the compiler's structured-reasoning advantage collapses; this null finding is informative as it bounds the claim to large-class instruction-tuned models and rules out artifacts specific to any single backbone.

**Table 10:** R7 multi-model robustness: compiled-vs.-unstructured cooperation gap across five LLM backbones.

Model	compiled	unstructured	Gap
Llama-3.3-70B-Instruct	0.828	0.764	+0.064
Qwen3-235B-A22B-Instruct	0.836	0.764	+0.072
Hermes-4-70B	0.823	0.764	+0.059
DeepSeek-V3.2	0.824	0.764	+0.060
Llama-3.1-8B-Instruct	0.775	0.764	+0.011

*n* = 80, BA, seed = 0.

The 8B-vs.-70B gap (+0.064−+0.011 = +0.053 in compiled cooperation) localizes the compiler's value within model capability: the compilation step requires a model that can reason coherently about a structured policy schema, propose targets and themes from a discrete vocabulary, and assess intensity calibration. Smaller models produce parseable IPL output (no solver fallbacks across any of the R7 runs) but converge on weaker policies. The exact location of the capability threshold is left for future work; we note only that it lies below 70B parameters and above 8B for the model families tested.

### 4.14 Non-stationary adaptation: Where the LLM's adaptive value emerges

Sections 4.6–4.8 established that under stationary conditions the LLM compiler is statistically indistinguishable from a hand-coded rule with identical parameters. We now ask: is there *any* regime in which the LLM's contribution exceeds rule equivalence? We test two perturbation classes: parametric (R8) and structural (R8b).

#### 4.14.1 R8: Parametric shock

At *t* = 50, the prosociality vector is multiplicatively reduced by 0.5 to model a sudden cooperation collapse. Two conditions, compiled (which re-deploys at every Δ*t* = 10 and may in principle propose a different policy in response to the shock-altered state snapshot) and static_hub_full (which deploys the frozen 〈hubs, hybrid, 0.55,periodic, 0.80〉 policy at every step), are compared across five seeds. We compute phase-resolved cooperation: pre-shift (*t* < 50), at-shock (*t* ∈ [50, 54]), recovery (*t* ≥ 60), and late (*t* ≥ 80).

The compiler's adaptive value is *not* detectable under parametric shock. Phase-resolved cooperation is statistically identical between compiled and static_hub_full in every phase (paired Mann–Whitney all *p* > 0.5, |*d*| ≤ 0.11 in post-shock phases; the recovery-phase gap is +0.001). The compiler keeps proposing the canonical policy (target stays hubs, theme stays hybrid, intensity wobbles within ±0.02 of 0.55 across seeds) regardless of the shock; recovery dynamics are dominated by the (unchanged) diffusion and reinforcement system. The honest interpretation is that the converged policy is *robust* to substantial parametric perturbations rather than *adaptive* to them, and this robustness is a property of the policy itself, not of the LLM that proposed it.

#### 4.14.2 R8b: Structural shock

The picture changes under structural perturbation. At *t* = 50, the BA graph is replaced with a freshly generated BA graph (different seed, same *n* and *m*) so that hub identity changes; mean hub overlap between the old and new top-9 degree sets is 0.58 across eight seeds, ranging from 0.44 to 0.67. compiled re-resolves the abstract token hubs against the *current* graph at each deploy event, while static_hub_full's frozen target list (the top-9 degree set computed at *t* = 0) keeps blasting former hubs that are no longer central. The two conditions are paired by seed (identical agent initialization, prosociality, susceptibility, and RNG sequence; only target resolution differs), enabling paired statistical tests with substantially higher power than unpaired comparisons.

Table 11 reports the phase-resolved paired analysis. In pre-shift and at-shock phases, no gap is detectable (3/8 seeds positive, paired *t* *p* > 0.1), as expected, no shock has yet differentiated the conditions. In recovery and late phases, *all eight seeds* align positively (paired Wilcoxon *p* = 0.004, the unanimous-direction floor at *n* = 8; paired *t* *p* = 0.0024 recovery, *p* = 0.0004 late; paired Cohen's *d* = 1.64 recovery, *d* = 2.27 late). Under Bonferroni correction with *m* = 4 phases, both post-shock results retain *p*_Bonf_ < 0.01. The hub-overlap-vs.-gap correlation provides a mechanism check: *r*_Spearman_ = −0.72 for recovery and −0.69 for late (both *p* ≈ 0.05 at *n* = 8); seeds with greater structural disruption (lower hub overlap) show larger compiled-vs.-static gaps.

**Table 11:** R8 (parametric shock) and R8b (structural shock) phase-resolved cooperation: paired analysis of compiled vs. static_hub_full.

Phase	R8 (prosocial shock)		R8b (graph rewire)	
	**Gap**	*d*_paired_	**Gap**	*d*_paired_
pre-shift (*t* < 50)	+0.002	0.38 (ns)	+0.001	0.57 (ns)
at-shock (*t* ∈ [50, 54])	+0.001	0.05 (ns)	+0.001	0.20 (ns)
recovery (*t* ≥ 60)	+0.001	0.11 (ns)	+0.005	1.64^**^
late (*t* ≥ 80)	+0.001	0.06 (ns)	+0.006	2.27^***^

R8 with five seeds, R8b with eight seeds. *d*_paired_ is paired Cohen's *d*; significance markers report Bonferroni-corrected paired *t* (*m* = 4).

^**^Paired Wilcoxon *p* = 0.004, paired *t* *p* = 0.002, *p*_Bonf_ < 0.01 (*m* = 4). ^***^Paired Wilcoxon *p* = 0.004, paired *t* *p* < 0.001, *p*_Bonf_ < 0.01 (*m* = 4). Bold values indicate statistically significant paired effect sizes after Bonferroni correction (*p*_Bonf_ < 0.01, *m* = 4).

**Figure 12:**
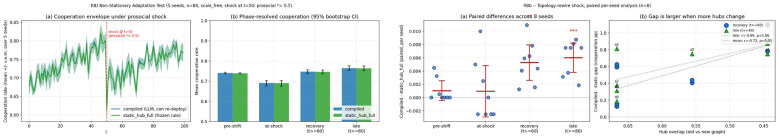
Non-stationary adaptation. **(Left)** R8, parametric shock (prosocial × 0.5 at *t* = 50): **(a)** cooperation envelope; **(b)** phase-resolved cooperation (95% bootstrap CI). compiled and static_hub_full are statistically indistinguishable: the canonical policy is robust to parametric perturbation. **(Right)** R8b, structural shock (BA graph replaced at *t* = 50, mean hub overlap 0.58): **(a)** paired differences across 8 seeds; **(b)** gap vs. hub overlap. compiled significantly outperforms static_hub_full in recovery and late phases (paired Wilcoxon *p* = 0.004, paired *t* *p* < 0.001, *d*_paired_ = 2.27 late); the *r*_Spearman_ = −0.72 correlation supports the structural-disruption mechanism.

Figure 12 contrasts the two perturbation classes side-by-side: the parametric shock (left) leaves the conditions overlapping, while the structural shock (right) opens a clean separation that all eight seeds replicate.

#### 4.14.3 Interpretation

The compiled-vs.-static gap under topology rewire is small in absolute terms (+0.6 pp at steady state) but statistically robust (*d*_paired_ = 2.27, *p* < 0.001), located precisely in the regime where the stationary-condition rule equivalence breaks down. Notably, the compiler does not adapt by changing its policy parameters: across all eight seeds, target_mode stays hubs, theme stays hybrid, and intensity wobbles within ±0.02 of 0.55 throughout. The advantage comes from the architectural property that hubs is a re-resolvable abstract token rather than a frozen node list. When the network's degree structure changes, the compiler's policy automatically retargets the new hubs at the next deploy event; the static rule, by construction, cannot.

The LLM compiler's contribution is therefore best characterized as *re-deployment with state-aware target resolution*, not real-time policy parameter optimization (which R8 rules out): its value emerges precisely when the network's structural assumptions shift. This property is generic to the abstract-policy paradigm and would also be exhibited by a non-LLM compiler that emitted IPL-conformant policies; the LLM's specific role is to provide such a compiler that requires no hand-coding of the policy generation logic and that, in principle, can propose substantively different policies under sufficient state change. In our experiments the compiler does not exercise this capacity, but the architecture preserves it.

### 4.15 Ablation summary

**Figure 13:**
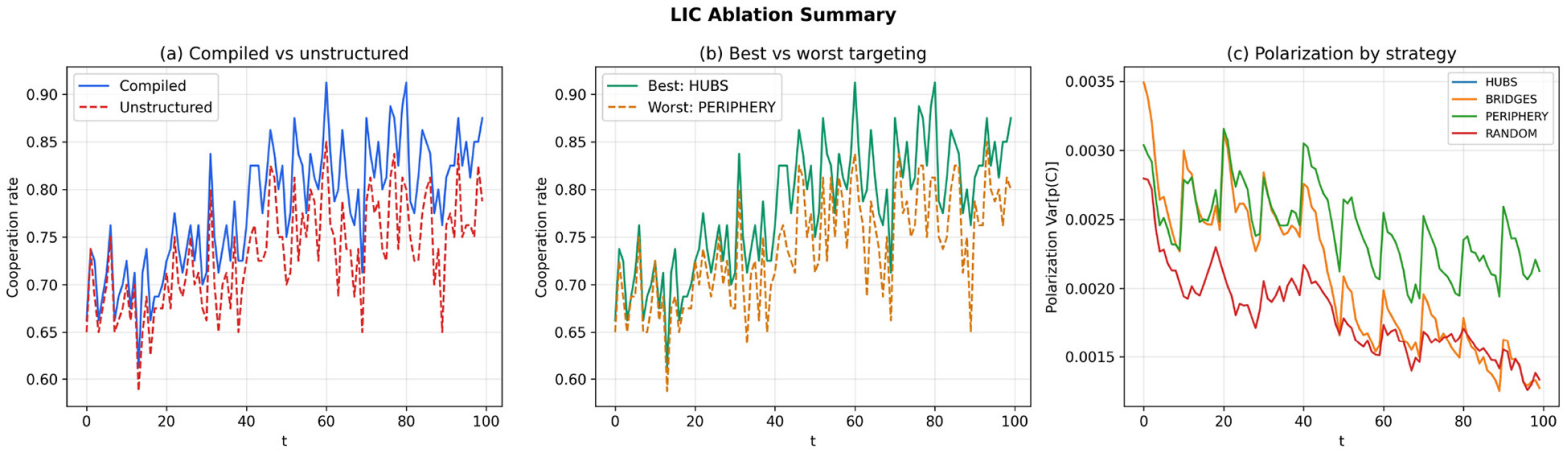
LIC ablation summary. **(a)** Compiled vs. unstructured: the compilation gap widens after *t* ≈ 30. **(b)** Best (hubs) vs. worst (periphery) targeting: 4.9 pp gap in final cooperation. **(c)** Polarization trajectories by strategy: periphery converges to substantially higher polarization (0.0022) than the other three strategies (~0.0014).

Figure 13 provides a consolidated three-panel ablation view.

Panel (a) recapitulates the compilation advantage: the compiled trajectory separates from the unstructured baseline around *t* = 30 and maintains a ~6 pp gap through steady state. Panel (b) confirms that the best-to-worst targeting gap is comparable in magnitude to the compilation gap itself, suggesting that intelligent targeting is responsible for a substantial portion of the compiler's advantage. Panel (c) shows that periphery is the only strategy that fails to converge to the low-polarization regime, remaining elevated at approximately 0.0022 while all other strategies descend below 0.0015 by *t* = 70.

## 5 Discussion

The central result of this paper, that compiled influence outperforms unstructured influence by 6.6 ± 1.0 percentage points across seeds, decomposes into three distinct mechanisms operating at different timescales. *Dose calibration* and *network-aware targeting* together account for ~95% of the gap and are matched by a hand-coded rule with the same parameters under stationary conditions (R2: *d*_compiled vs. static_ = 0.18, ns; Section 4.8). The LLM's role under stationarity is therefore best characterized as *automated rule discovery and constraint enforcement*: the solver–critic pipeline reliably converges on a near-optimal parameterization of the IPL space (R7: 4/5 large backbones agree on the converged parameters; Section 4.13) and the critic enforces format and risk constraints that we demonstrate are functional on adversarial inputs (R6-EXT: 20/20 risky policies flagged high and rejected; Section 4.12). The remaining contribution, where the LLM's value cannot be substituted by a frozen rule, is *architectural*: under structural non-stationarity (R8b; Section 4.14), the LLM-mediated re-deployment loop produces a +0.6 pp advantage over static deployment with *d*_paired_ = 2.27 (*p* < 0.001), scaling negatively with hub overlap. This advantage emerges not because the LLM proposes different policy parameters, but because its abstract policy language (hubs) re-resolves against the current network at each deploy, while a one-shot static deployment cannot.

The 7.4× asymmetry between targeting and framing contributions is perhaps the most actionable finding. It suggests that for practitioners designing LLM-mediated influence systems, investing in network-aware targeting infrastructure will yield far greater returns than investing in sophisticated narrative generation. A simple hub-targeting strategy with a mediocre narrative substantially outperforms a random-targeting strategy with an optimal narrative. This finding also has implications for adversarial settings: defenders concerned about malicious influence should prioritize monitoring *who* is being targeted over analyzing *what* is being said.

The compiler's compensatory behavior under theme ablation, boosting intensity for weaker multipliers, reveals an emergent form of closed-loop control. The solver observes the population state snapshot (which implicitly reflects the theme's effectiveness through cooperation rates and exposure levels) and adjusts its proposed intensity accordingly. This behavior was not explicitly programmed but arises from the LLM's ability to reason about the relationship between policy parameters and societal outcomes.

### 5.1 Practical implications

Beyond the theoretical analysis, the LIC framework suggests concrete design guidelines for real-world multi-agent coordination systems. First, our results indicate that allocating engineering effort to structural targeting mechanisms yields substantially larger performance gains than investing in narrative sophistication: a simple hub-targeting strategy with a generic framing outperforms random targeting with an optimal narrative by approximately 5 percentage points. This insight applies directly to recommender systems, decentralized coordination platforms, and organizational communication tools where influence budgets are finite. Second, the hub–bridge equivalence in scale-free topologies implies that lightweight degree-based targeting (*O*(*n*)) can replace expensive betweenness computation (*O*(*nm*)) without performance loss, a practical advantage at scale. Third, the compiler's structured output format enables mechanical compliance checking: governance layers can inspect, constrain, and audit each policy field independently before deployment, a capability that is impossible when influence is mediated through unconstrained natural-language generation. These properties make the LIC architecture a candidate for settings where influence deployment must be simultaneously efficient, transparent, and ethically bounded, such as public health campaigns on social networks, corporate change management, or multi-stakeholder negotiation platforms.

### 5.2 The architectural locus of LLM value

A natural question motivated by the multi-seed equivalence between compiled and static_hub_full is: why use an LLM at all if a hand-coded rule matches it under stationary conditions? Our R8b result answers this precisely. The LLM's value is neither in real-time policy optimization (which R8 rules out) nor in proposing parameters that a domain expert could not derive (R1's decomposition shows the canonical 〈hubs, hybrid, 0.55,periodic, 0.80〉 policy is reproducible by hand). The value is in the *coupling* between three architectural properties: (1) the compiler's abstract policy output, target_mode = hubs as a token rather than a frozen node list; (2) periodic re-deployment driven by a state snapshot; and (3) state-aware target resolution at deploy time. When the network's structure shifts mid-simulation, this loop automatically corrects the targeting; a one-shot static deployment cannot. The LLM is the proposing element of this loop because it can in principle propose other parameters under sufficient state change. In our experiments it does not, but the architecture preserves that capacity, which is a non-trivial property of the framework rather than an empirical claim about LLM optimization in real time.

The numerical identity of hubs and bridges conditions across all timesteps and metrics was unexpected and warrants discussion. In Barabási–Albert networks, preferential attachment ensures that early-arriving nodes accumulate both high degree and high betweenness centrality, because they sit on many shortest paths precisely because they have many connections (Barabási and Albert, 1999). This structural correlation is well-known in network science (Newman, 2003), but its operational consequence for influence targeting has not been explicitly demonstrated in the LLM-agent setting: the *O*(*n*) degree-based targeting is a perfect substitute for *O*(*nm*) betweenness computation. We note that this equivalence is topology-dependent and would likely break in networks with community structure (e.g., stochastic block models) where high-betweenness bridge nodes may have moderate degree but connect otherwise-separated clusters.

The finding that hub-targeted influence produces *lower* final exposure inequality than random or periphery targeting, despite starting with higher initial inequality, reveals a non-obvious dynamic. The explanation lies in the interaction between the gradient-based diffusion mechanism and hub connectivity. When a hub receives a large dose, the exposure gradient between it and its many low-exposure neighbors is steep, driving rapid outward diffusion. As neighbors' exposure rises, the gradient flattens, and the hub's own exposure decays through fatigue, gradually equalizing the distribution. Periphery targeting lacks this self-correcting mechanism: a peripheral node with 2–3 neighbors generates only a weak gradient, and the dose remains trapped in a local neighborhood.

This dynamic has implications for fairness in influence systems. Naively, one might impose equity constraints that prevent targeting high-degree nodes, reasoning that concentrated influence is inherently unfair. Our results suggest the opposite: hub targeting achieves *both* higher cooperation *and* better exposure equity at steady state, because the network's diffusion dynamics redistribute the initial concentration.

### 5.3 Threats to validity

#### 5.3.1 Internal validity

Several design choices affect the causal claims within our experimental setting. Multi-seed replication (R2, *n* = 5 seeds) confirms statistical significance under Bonferroni correction for the headline compiled-vs.-unstructured comparison (Section 4.8); theme-ablation differences fall below this threshold and are now reported as null findings. The solver–critic pipeline converges to a single canonical policy across all 360 production deploy events in R1–R5; this stability holds even under the prosocial and structural shocks of R8 and R8b, suggesting the convergence is robust within the parameter regime tested but may not generalize to more adversarial or volatile conditions. The theme effect multipliers (1.05–1.20) are stipulated rather than empirically calibrated against human persuasion data; wider multiplier ranges could yield a different targeting-to-framing attribution ratio. Critic gating is verified responsive on adversarial inputs (R6-EXT) but is not exercised by the solver in production, which is a property of the solver rather than a limitation of the critic.

#### 5.3.2 External validity

The generalizability of our findings is constrained along several dimensions. Topology dependence (R3) tests four classes (BA, WS, ER, modular SBM) and confirms the compiled advantage in all of them; the magnitude is largest in BA and smallest in WS, consistent with the prediction that targeting leverage scales with degree heterogeneity. Network size (R4) ranges from *n* = 80 to *n* = 640 with the gap stable at ~+6 pp; the targeting advantage thus scales an order of magnitude beyond the primary experimental size. Multi-model replication (R7) demonstrates the result is not specific to a single LLM backbone. Real-network validation, with continuous-time tie evolution and heterogeneous agent-level responses to influence, remains future work. Most critically, the numerical agents represent an idealized setting in which agents respond deterministically to the influence dose through a known logistic function. The term “agent” in our framework is model-agnostic, it encompasses LLM-powered agents, rule-based entities, or human participants, but the reader should not conclude that the reported cooperation gains transfer directly to practice. Real agents may selectively ignore instructions, reinterpret framing, or exhibit resistance dynamics absent from our logistic model. Validating the compilation advantage under realistic agent architectures, with their inherent limitations in instruction following, state reporting, and persuasion susceptibility, is a priority for future work.

#### 5.3.3 Construct validity

Our primary metric, the cooperation rate in an iterated public-goods game, captures one dimension of collective behavior but does not exhaust the space of desirable multi-agent outcomes. Alternative constructs such as social welfare, individual utility variance, trust formation, or communication quality might respond differently to compiled vs. unstructured influence. The polarization measure (Var[*p*(C)]) captures distributional spread but not ideological clustering or opinion fragmentation, and the Gini coefficient of exposure measures distributional equity but not whether the *right* agents (e.g., those most in need of coordination support) receive influence. Future work should evaluate the compiler against a broader set of outcome constructs to assess whether the structural advantages demonstrated here generalize beyond cooperation rate.

#### 5.3.4 Connection to constitutional governance

The compiler abstraction developed in this paper creates both an opportunity and a risk. The opportunity is that structured policies can be *governed*: a constitutional layer can inspect each compiled policy before deployment, rejecting policies that exceed intensity thresholds, use forbidden themes, or produce exposure distributions that violate equity constraints. The risk is that the same compiler could be used to optimize influence for manipulation, maximizing compliance while minimizing detection, if no governance layer is in place. In companion work, we develop a constitutional governance framework that addresses this risk by imposing hard constraints (reject violating policies) and soft optimization (penalize manipulation risk in the selection utility), ensuring that the cooperation gains demonstrated here do not come at the cost of agent autonomy or epistemic integrity.

## 6 Conclusion

We have introduced the LLM Influence Compiler (LIC), a framework that formalizes the mapping from natural-language influence directives to structured, parameterized policies in multi-agent societies. The Influence Policy Language defines a five-field schema over which a solver–critic pipeline compiles population state snapshots into executable policies with explicit targeting, framing, intensity, timing, and confidence parameters.

Across nine controlled experiment blocks comprising more than 200 simulation runs, spanning four topologies (Barabási–Albert, small-world, Erdős–Rényi, modular SBM), four network sizes (*n* ∈ {80, 160, 320, 640}), and five LLM backbones, compiled policies raise the mean cooperation rate to 0.826 ± 0.010 (95% bootstrap CI [0.819, 0.834]), an 8.6% relative improvement over the unstructured baseline (0.760 ± 0.005, Mann–Whitney *p*_Bonf_ = 0.040, Cohen's *d* = 7.90), while simultaneously achieving faster diffusion, lower polarization, and superior exposure equity. Controlled ablations reveal that network-position targeting accounts for 7.4× more cooperation variation than narrative framing, a finding that is only visible through the structured decomposition afforded by the compiler abstraction. Hub and bridge targeting dominate the Pareto frontier of speed vs. polarization, and the compiler exhibits emergent compensatory behavior, adjusting intensity to offset weaker theme multipliers. Under structural non-stationarity (mid-simulation graph rewire), the LLM-mediated re-deployment architecture significantly outperforms a frozen rule with identical static parameters (paired *t*-test *p* < 0.001, paired Cohen's *d* = 2.27, 8/8 seeds), demonstrating that the compiler's adaptive value emerges precisely when network structural assumptions shift.

Beyond effectiveness, the compiler's primary contribution is architectural: by converting an opaque generative process into a decomposable, auditable policy object, it enables the independent attribution, constraint, and governance of each influence component. This lays the foundation for constitutional oversight frameworks that ensure LLM-mediated influence is not only effective but also transparent and ethically bounded.

Remaining future directions include (1) deployment on networks with continuous-time edge evolution and node birth/death dynamics, (2) closing the loop with LLM-powered agents to test compiler–agent interaction effects, (3) extending the constitutional governance layer (companion work) to handle multi-objective ethical constraints, and (4) characterizing the model-capability threshold (R7's 8B-vs.-70B gap) more precisely across model families and parameter scales.

## Data Availability

The datasets presented in this study can be found in online repositories. The names of the repository/repositories and accession number(s) can be found below: https://github.com/drdezarza/lic.
